# Social and behavioral risk reduction strategies for tuberculosis prevention in Canadian Inuit communities: a cost-effectiveness analysis

**DOI:** 10.1186/s12889-021-10187-z

**Published:** 2021-02-03

**Authors:** Aashna Uppal, Olivia Oxlade, Ntwali Placide Nsengiyumva, Dieynaba S. N’Diaye, Gonzalo G. Alvarez, Kevin Schwartzman

**Affiliations:** 1 Montreal Chest Institute, Montreal, Quebec Canada; 2 Respiratory Epidemiology and Clinical Research Unit, Centre for Outcomes Research and Evaluation, Research Institute of McGill University Health Centre, Montreal, Quebec Canada; 3 McGill International Tuberculosis Centre, 1001 boulevard Décarie, Room D05.2511, Montréal, Québec H4A 3J1 Canada; 4 Department of Epidemiology, Biostatistics and Occupational Health, McGill University, Montreal, Quebec Canada; 5 The Ottawa Hospital Research Institute, Department of Medicine, Division of Respirology, The Ottawa Hospital, University of Ottawa, Ottawa, Ontario Canada

**Keywords:** Cost-effectiveness, Decision analysis, Tuberculosis, Tobacco, Alcohol, Food insecurity, Overcrowding, Inuit, Nunavut

## Abstract

**Background:**

Tuberculosis (TB) is an important public health problem in Inuit communities across Canada, with an annual incidence rate in 2017 that was nearly 300 times higher than in Canadian-born non-Indigenous individuals. Social and behavioral factors that are prevalent in the North, such as commercial tobacco use, excessive alcohol use, food insecurity and overcrowded housing put individuals at higher risk for TB morbidity and mortality. We examined the potential impact of mitigation strategies for these risk factors, in reducing TB burden in this setting.

**Methods:**

We created a transmission model to simulate the epidemiology of TB in Nunavut, Canada. We then used a decision analysis model to assess the potential impact of several evidence-based strategies targeting tobacco use, excessive alcohol use, food insecurity and overcrowded housing. We predicted TB incidence, TB-related deaths, quality adjusted life years (QALYs), and associated costs and cost-effectiveness over 20 years. All costs were expressed in 2018 Canadian dollars.

**Results:**

Compared to a status quo scenario with no new interventions for these risk factors, the reduction strategy for tobacco use was most effective and cost-effective, reducing TB incidence by 5.5% (95% uncertainty range: 2.7–11%) over 20 years, with an estimated cost of $95,835 per TB case prevented and $49,671 per QALY gained. The addition of the food insecurity reduction strategy reduced incidence by a further 2% (0.5–3%) compared to the tobacco cessation strategy alone, but at significant cost.

**Conclusions:**

Strategies that aim to reduce commercial tobacco use and improve food security will likely lead to modest reductions in TB morbidity and mortality. Although important for the communities, strategies that address excess alcohol use and overcrowding will likely have a more limited impact on TB-related outcomes at current scale, and are associated with much higher cost. Their benefits will be more substantial with scale up, which will also likely have important downstream impacts such as improved mental health, educational attainment and food security.

**Supplementary Information:**

The online version contains supplementary material available at 10.1186/s12889-021-10187-z.

## Background

Tuberculosis (TB) remains the deadliest infectious disease worldwide, with 1.4 million deaths in 2019 [1]. It is often the most vulnerable people who bear a disproportionate burden of TB morbidity and mortality; this is the case with Indigenous peoples [2]. This reflects colonization and disenfranchisement, as well as a high prevalence of risk factors for TB in many Indigenous communities, such as Aboriginal peoples and Torres Strait Islanders in Australia, Maori communities in New Zealand, and Inuit, First Nations and Métis communities in Canada. These risk factors include diabetes, alcohol and other substance use, cigarette smoking, food insecurity, and overcrowded housing [2].

Canada is among the countries with the lowest overall TB incidence. However, the disease is concentrated in Canadian Indigenous communities, particularly in the North [3]. TB was introduced to the Eastern Canadian Arctic in the 1800s following European settlement in the region [4]. After a steady decline over the latter half of the twentieth century, there has been a recent resurgence of TB in Inuit communities across Canada, with an annual incidence rate in 2017 that was 400 times higher than in Canadian-born non-Indigenous individuals (an incidence rate of 4.9 per 100,000 in Canada, 0.5 per 100,000 in Canadian-born non-Indigenous individuals, and 205.8 per 100,000 in Inuit communities) [4, 5]. In this context, it is important to consider the role of social and behavioural factors, in addition to other biological or environmental factors, that put individuals at higher risk for TB morbidity and mortality.

Our study focuses on Nunavut, the largest region of the Inuit Nunangat (Inuit homeland). We consider four highly prevalent social and behavioral risk factors for TB: commercial tobacco use, heavy alcohol consumption (which we refer to as heavy drinking), food insecurity, and overcrowded housing, all of which are associated with increased risk of TB, and some with poorer outcomes. In 2014, Nunavut’s population aged over 12 had the highest proportion of commercial tobacco smokers of all Canadian provinces and territories, with 62% estimated to be current smokers [6]. Although 14% reported heavy drinking, similar to 16% in the rest of Canada, there is likely underreporting because of legal restrictions and contraband use [6, 7]. In 2012, 56% of Nunavut households were food insecure, and in 2016, the same percentage of individuals lived in overcrowded dwellings (defined as more than one person per room) [8, 9] compared to 8% food insecure households and 2% in overcrowded dwellings, in the rest of Canada [9–11].

These risk factors affect TB pathogenesis in different ways. Smoking is associated with substantially increased risk of acquiring TB infection, progression to TB disease and TB-related death, possibly as the result of impaired immunity [11–13]. Excessive alcohol use is associated with increased susceptibility to TB infection and disease, and poorer treatment outcomes due to suboptimal adherence [14, 15]. Food insecurity increases the risk of TB infection and poor treatment outcomes such as treatment failure and death [16, 17]. Finally, crowded housing is associated with a higher contact rate between individuals, thereby increasing the likelihood of *M. tuberculosis* transmission [18–20]. There exist culturally appropriate programs to address these risk factors; for example, plain packaging was introduced for commercial tobacco products in Aboriginal communities in Australia [2]. Similarly, in several Nunavut communities, an inpatient program focused on clinical and cultural healing is designed to reduce heavy drinking [21].

Current approaches to TB prevention, detection and care in Nunavut include (but are not limited to) screening, contact investigation, active surveillance, molecular diagnostics such as the Xpert® MTB/RIF test, and case management in accordance with the Canadian Tuberculosis Standards [22]. On the other hand, the current TB program does not specifically address social and behavioral risk factors for TB. Given their high prevalence in Nunavut and their association with TB morbidity and mortality, our objective is to assess the contribution of programs that reduce their prevalence. Specifically, we use simulation modeling to estimate the potential impact of several evidence-based strategies targeting four risk factors, considering TB-related health outcomes, associated costs and cost-effectiveness over 20 years from the government payer perspective.

## Methods

We built two simulation models. The first, a dynamic transmission model, simulated the historic and recent TB epidemics in Nunavut, in order to estimate the distribution of TB-related health states in the general population in 2018 (e.g. the proportion of the population susceptible to TB, the proportion latently infected, as these are not directly measured or known). The second was a decision analysis model, which involved using the 2018 population distribution (obtained from the dynamic model) to then evaluate the cost-effectiveness of various risk factor reduction strategies over the following 20 years. Each model is described in further detail below.

### Dynamic model

A dynamic transmission model was first created and validated to simulate the TB epidemic in Nunavut from 1948 to 2018. This model’s structure was adapted from a previously published model [23]. The previous model only considered smoking as a risk factor; we integrated three additional risk factors: excessive alchol use, food insecurity and overcrowded housing. This model captured the pre-antibiotic period, the shift in Inuit living conditions, the introduction of antibiotics, and recent prevention and care measures. Model parameters were based on published literature, whilst unknown parameters were based on observed data. By simulating the spread of TB in this region, we were able to estimate the distribution of the population with respect to the four risk factors as well TB-related health states in 2018, which included the proportion of the population uninfected by *Mycobacterium tuberculosis*, the proportion of the population with latent infection, and proportion of the population who had recovered from active disease. Pathogenetic parameters that were calibrated in the dynamic model were also applied to the decision analysis model (see Additional file 1, which also provides more details of the dynamic model).

### Decision analysis model

**Fig. 1:**
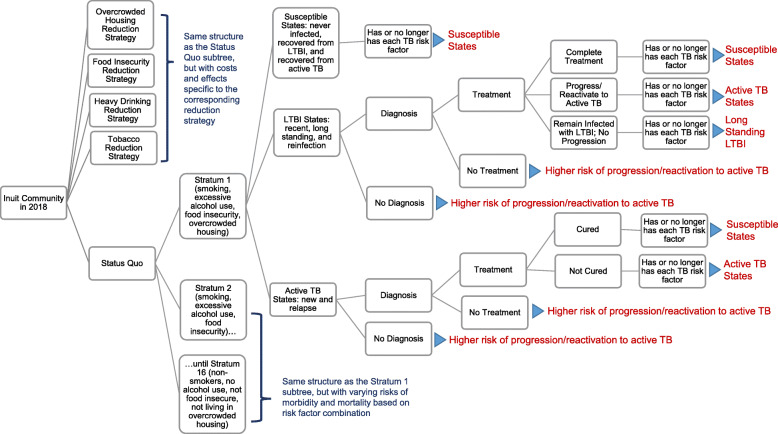
Simplified schematic of decision analysis model structure. * At the end of each cycle, individuals either continue to have or stop having any of the four TB risk factors (tobacco use, heavy drinking, food insecurity, overcrowded housing). Whether they continue or stop depends on how the prevalence of the factors is changing. For example, if an individual in Stratum 1 (i.e. with all four TB risk factors) is no longer in overcrowded housing by the end of one cycle, they move to Stratum 2 (i.e. having all TB risk factors except overcrowding) at the beginning of the following cycle

Using TreeAge Pro software (TreeAge Software Inc., 2018, Williamstown, MA), we created a Markov decision analysis model which simulated a cohort of Canadian Inuit with median age 20 [24], starting in 2018 [25, 26]. As with the dynamic model, the model structure was modified from a previous version that considered only smoking as a risk factor [23]; a simplified schematic presentation of its structure is shown in Fig. 1. This model was used to predict TB-related health outcomes and TB-related health system costs over a 20-year period after the implementation of the risk factor reduction strategies, compared with a status quo scenario without the implementation of these specific risk factor reduction strategies. The status quo scenario incorporated the current standard of TB care in the region. The health outcomes considered were TB incidence, TB-related deaths and TB-related quality adjusted life-years (QALYs). A discount rate of 3% was applied to all future outcomes and costs [27].

### Data used in the decision analysis model

Parameters used in the decision analysis model fell into four main categories: (1) TB pathogenetic and epidemiologic parameters, (2) TB-related health utilities, (3) TB risk factor reduction strategies’ costs and effects, and (4) TB-related health system costs.

TB pathogenetic and epidemiologic parameters were informed by published data, or where necessary were calibrated using our dynamic model. Systematic reviews and meta-analyses were used to inform epidemiologic parameters describing the effect of each social and behavioural risk factor on TB pathogenesis. TB-related health utilities generally also came from published literature. On the other hand, data related to the costs and effects of TB risk factor reduction strategies came from actual program data, where possible, as described below. Finally, TB-related health system costs reflected publications from Nunavut, or other comparable settings. We distinguish two types of costs used in the model: those related to risk factor reduction strategies and those related to TB care (which we refer to as TB-related health system costs). [A full list of model parameters along with specific data sources is provided in Additional file 1 – Supplemental Table 5.]

#### TB pathogenetic and epidemiologic parameters

Table 1 shows the key decision analysis model parameters related to TB pathogenesis, epidemiology and treatment. These parameters were used to define the probability of events occurring in the model (as one-time percentages, unless otherwise stated). For the population exposed to a combination of risk factors, the values for the relevant parameters were multiplied by the corresponding estimates of effect for the risk factors (Table 2).

**Table 1:** Decision analysis model parameters related to the natural history, epidemiology, treatment of LTBI and TB

ESTIMATE	PROBABILITY (%) or VALUE (RANGE)	REFERENCE
**Probabilities (once-time percentages)**		
Probability of completing latent TB infection treatment among those who initiate treatment	75.6	[28]
Probability of diagnosing active disease	90	Assumption
Probability of spontaneous resolution of untreated TB disease	25	[29]
Probability of relapse after active TB treatment	1.4	Dynamic model^b,d^
Protective immunity from previous TB disease	55	Dynamic model^b^
**Probabilities for those without any of the four specific TB risk factors**		
Probability of rapid progression following TB infection	0.18/year^a^	Dynamic model^b^
Probability of reactivation of longstanding latent TB infection to TB disease	0.05/year	[30–32]
Probability of dying from untreated TB	7.7/year^c^	[33]
Probability of dying from TB during treatment	1.7/year^c^	[34]
**Quality Adjusted Life Years for Latent TB Iinfectuin and active TB**		
Utility score for individual with active TB disease (during treatment)	0.85 (0.70–0.90)	[35]
Utility score for individual with active TB disease (before treatment)	0.68 (0.65–0.72)	[36]
Utility score for individual with latent TB infection, (during treatment)	0.97 (0.95–1.00)	[36]
Utility score for individual with latent TB infection (before treatment)	1	Assumption^e^

^a^*This value is very low because it applies only to those not exposed to any of the four TB risk factors. Taking a weighted average across the 16 strata gives a rapid progression value of 0.8%/year*

^b^*Calibrated using dynamic model, see Additional file* *1**– section “Calibration details”*

^c^*Taking a weighted average of these values over all 16 strata gives values from literature*

^d^*Although this is calibrated, its initial value was based on the literature: the probability of relapse following active TB treatment started at 1.5%* [34, 37]

^e^*Individuals with latent TB infection are asymptomatic and are assumed to be otherwise healthy*

**Table 2:** TB risk factor specific multipliers applied to probabilities in decision analysis model

Outcome	MEASURE of EffecT^a^	ESTIMATE (95% CI)	Comparison	Reference
SMOKING				
Infection	Relative Risk	1.9 (1.6–2.3)	Ever or current smokers vs. never smokers	[11]
Active Disease	Relative Risk	1.5 (1.3–1.7)	Ever or current smokers vs. never smokers	[12]
Death	Relative Risk	2.6 (1.8–3.6)	Ever or current smokers vs. never smokers	[11]
HEAVY DRINKING				
Infection	Relative Risk	2.9 (1.9–4.6)	≥40 g alcohol per day and/or alcohol use disorder vs. < 40 g alcohol per day and no alcohol use disorder	[38]
Active Disease	Relative Risk	4.2 (2.7–6.5)	≥40 g alcohol per day and/or alcohol use disorder vs. < 40 g alcohol per day and no alcohol use disorder	[39]
Death	Hazard Ratio	2.4 (1.1–5.3)	Addiction to alcohol vs. no addiction to alcohol^**#**^	[40]
FOOD INSECURITY				
Infection	Odds Ratio	2.1 (1.0–4.3)	Inadequate daily fruit/vegetable intake vs. adequate daily fruit/vegetable intake^b^	[16]
Active Disease	Odds Ratio	2.4 (1.6–3.1)	People experiencing household food shortage vs. not experiencing household food shortage	[41]
OVERCROWDING				
Infection	Relative Risk	1.5 (1.1–2.0)	Houses with > 1 person per room vs. houses with ≤ 1 person per room	[20]
Active Disease	Odds Ratio	1.3 (1.2–1.5)^c^	For every additional 0.2 persons per room	[19]

^a^Where possible, we obtained adjusted estimates from each study

^b^Adequacy of daily fruit and vegetable intake was determined based on North American reference standards for age and sex, assuming individuals had a “typical” level of physical activity

^c^We modified this estimate to reflect the added risk for every additional 1 person per room (we used 1.3^5^ = 3.7 [95 % *CI* 2.5 − 7.6])

#### TB-related health utilities

In order to estimate QALYs associated with TB, we used TB-related health utility weights from 0 to 1 (where 0 is death and 1 is perfect health) [42]. Scores were calculated based on the length of time spent with or without active TB or LTBI treatment. As latent TB is asymptomatic by definition, we considered that persons with LTBI who are untreated have the same health utility value as persons in perfect health, i.e. a value of 1. We did not consider utility weights associated with TB risk factors (e.g. food insecurity, overcrowded housing). Table 1 shows the specific utility values used to estimate QALYs.

After adjusting pathogenetic parameters for persons with social and behavioral risk factors for TB, all effect estimates for the four risk factors reflected the published literature, and wherever possible these were independent estimates (i.e. adjusted for other potential risk factors). For example, for those who were at increased risk due to smoking, the annual probability for rapid progression following newly acquired TB infection shown in Table 1 (i.e. 0.18% for those not at risk for any specific TB risk factor) was multiplied by the estimate of effect for active TB conditional on infection (RR = 1.5) to give a higher probability of rapid progression for smokers (0.18% × 1.5 = 0.27% for those at risk due to smoking). Similarly, for those at increased risk due to both smoking and heavy drinking, the annual probability of rapid progression following TB infection was 0.18% × 1.5 × 4.2 = 1.13%.

#### TB risk factor reduction strategies

We considered multiple risk reduction strategies. Each strategy selected was highlighted by community members, public health, and academic informants in Nunavut, and wherever appropriate, was a land-based Inuit-led intervention. Land-based strategies were those that incorporated the land in their structure, rooted in the notion that the land is a fundamental component of health, wellbeing and culure for Indigenous Peoples [43]. A systematic review of the proximate determinants of TB in Indigenous communities [2] helped inform potential risk reduction strategies. In instances where we were unable to identify strategies implemented in the Arctic, we used information from other relevant settings. In our final short list, we considered only those reduction strategies where information was available on both cost and effect, and which were considered acceptable by informants in Nunavut. Cost and effect data often came from separate reports. A brief summary follows. Detailed descriptions of each of the strategies selected are provided in Additional file 1 – section “Risk factor reduction strategies”.

#### Tobacco reduction strategy

Our previous analysis addressing tobacco reduction considered several cessation strategies separately, and in combination [23]. A combined strategy consisting of pharmacotherapy coupled with counselling, mass-media campaigns and increased tobacco taxation was found to be the most cost effective approach to reducing tobacco use. We therefore used this combined approach as the primary tobacco reduction strategy in our current analyses.

#### Heavy drinking reduction strategy

The heavy drinking reduction strategy is centred on a land-based healing camp [44]. Such a program currently operates out of the Cambridge Bay Wellness Centre, and is set to expand to two other regions in Nunavut [21]. The strategy assumes a 28-day inpatient camp offered 3 to 4 times per year, with programming focused on both clinical and cultural healing. For our analysis, we assumed the program was scaled up in the three regions (Kitikmeot, Kivalliq and Qikiqtaaluk) by a factor of 5, so as to achieve sufficient population coverage. Costs were scaled up proportionally, based on published budgets [21].

#### Food insecurity reduction strategy

We considered a comprehensive approach to reducing food insecurity that incorporated initiatives based in Nunavut and the Northwest Territories. Specifically, we considered as prototype programs the Niqinik Nuatsivik Nunavut Food Bank, the First Nations and Inuit component of the Canada Prenatal Nutrition Program (CPNP) [45], Nunavut’s Country Food Distribution Program (CFDP), and a community greenhouse project in Hay River, Northwest Territories in combination [46–49]. Based on the available information, costs were adjusted to reflect full coverage of the food-insecure population (or in the CPNP’s case, all food-insecure pregnant women).

#### Overcrowded housing reduction strategy

During 2014–2015, there were 210 housing units constructed in 12 communities in Nunavut and 216 housing units in 8 communities in Nunavik (the Inuit homeland within Quebec) [50]. These housing units were a combination of one-, two- and four-bedroom houses and apartments. We used data related to construction of these 426 units to inform the strategy for reducing overcrowded housing.

### Effects of mitigation strategies on associated risk factors

The anticipated effect of each mitigation strategy on the associated risk factor is summarized in Table 3. We obtained estimates of effect from the published literature which described the implementation of the selected strategies [50–54]. Additional details regarding the estimate of effect for risk factor reduction strategies are provided in Additional file 1 - Supplemental Table 4.

**Table 3:** Estimated impact of reduction strategies on TB risk factor prevalence

Background change in prevalence of risk factor without reduction strategy^a^	Estimate of effect of reduction strategy	Reduction strategy’s annual reach	Change in prevalence of risk factor with reduction strategy^b^
SMOKING			
Decrease in number of smokers by 0.013% per year	A systematic review indicates that persons receiving pharmacotherapy and counselling in communities receiving mass media interventions were twice as likely to quit commercial tobacco smoking than those who received usual care; Relative Risk = 2.36 (95% CI 1.01–5.50) [55]. A SimSmoke simulation notes that 25% price increase leads to 7% reduction in smoking prevalence within three years and increases over time to 14% [51, 56]	The entire smoking population is exposed to taxation plus the availability of pharmacotherapy and counselling (but only a fraction make quit attempts; see Table 4). Mass media campaigns, however, reach both smokers and non-smokers. All components of this combined strategy occur within the first year of the simulation, and do not repeat after that. Their effect, however, lasts longer [23].	Decreases the smoking population by 5.54% per year for the first 3 years, then by 1.01% per year for the next 7 years. Returns to background decrease of 0.013% per year for the remaining 10 years.
HEAVY DRINKING			
Increase in number of heavy drinkers by 0.07% per year	A cohort study in California exploring the effectiveness of a holistic cultural treatment program suggests that inpatient participants experience a 28.3% absolute reduction in use of alcohol and drugs in the 30 days following their treatment session (31.3% at baseline vs. 3.0% at follow-up) [54].	The mobile treatment center serves 8 individuals per session × 4 sessions × 3 regions = 96 individuals. In the model, this is scaled up by a factor of 5 (480 individuals ≈ 7% of the heavy drinking population). The 20 sessions in each region occur within the first year of the model, and do not repeat after that. As such, their effect also occurs within the first year of the simulation.	Decreases the number of heavy drinkers by 1.91% in the first year. Returns to background increase of 0.07% per year for the remaining 19 years.
FOOD INSECURITY			
Decrease in number of persons living with food insecurity by 0.36% per year	A case-control study conducted among 533 households in 14 communities in Northern Manitoba noted that the presence of a country food program increases the likelihood of being food secure; Odds Ratio = 20.64 (95% CI 2.42–176.08) [53]. Because of the uncertainty around the point estimate and its magnitude, we have used the lower bound of the 95% confidence interval as a conservative measure of impact, and investigated its effect further in sensitivity analysis.	All four initiatives in this strategy have been scaled up to reach the entire food-insecure population. The Canada Prenatal Nutrition Program, however, reaches only food-insecure pregnant women (≈2.3% of the food insecure population).	Decrease in the number of food insecure persons by 0.87% each year for all 20 years.
OVERCROWDING			
Increase in persons living in overcrowded housing by 2.27–5.76% per year^c^	The cohort study that our overcrowding reduction strategy is based on reports a 46% absolute reduction in overcrowding among those who were accommodated by the new housing units (65.5% at baseline vs. 19.5% at follow-up) [50].	426 housing units built × 3.3 individuals on average in each unit at follow-up = 1406 individuals (≈ 6% of the overcrowded population). The housing units are built in the first year of the model, but their associated maintenance recurs annually. The corresponding effect on overcrowding prevalence occurs within the first year of the simulation.	Decrease in persons living in overcrowded housing by 0.49% for the first year, reffecting this effect superimposed on background increase in overcrowding. Returns to background increase by 2.27–5.76% each year for the next 9 years, to reach 100% by year 10. No change in prevalence for the remaining 10 years^d^.

^a^These changes were informed by trends in TB risk factor prevalence over time in Nunavut

^b^Calculations illusrating how the estimates of effect translate into changes in prevalence are provided in Additional file 1 - Supplemental Table 4

^c^Overcrowding prevalence increases parabolically, not linearly, so the rate at which it increases does not remain constant (but remains between 2.27 and 5.76%)

^d^Overcrowding prevalence reaches 100% during the 10th cycle of the simulation, because of background population growth exceeding housing supply, so there is no additional increase after that

We assumed that the population that was no longer exposed to a particular risk factor would remain without it for the rest of the simulation. For example, those who were no longer food insecure as the result of the intervention were categorized as food secure for the remaining simulation. For all scenarios we also considered the ongoing background change in risk factor prevalence, in the absence of any specific new reduction strategy. The rate of background change was informed by published literature on risk factors from Nunavut [6, 8, 9, 57–63].

### Costs

All costs are expressed in 2018 Canadian dollars and are from the government payer perspective [64].

#### Costs related to social and behavioural risk factor reduction strategies

Table 4 summarizes the costs associated with each of the reduction strategies. A more detailed description of costs is available in Supplemental Table 5 within Additional file 1. Wherever possible, we used reported costs from Nunavut associated with each reduction strategy. When that was not possible, we integrated published costs from comparable settings. For example, tobacco reduction strategy costs reflected a Centers for Disease Control and Prevention (CDC) report and Régie de l’assurance maladie du Québec (RAMQ) data [65, 66]. Charges to buyers related to tobacco taxation do not represent net costs for government health payers or society as a whole. Costs for the healing camps (alcohol use reduction) were based on a report that outlined their implementation across Nunavut [21]. Costs related to the initiatives included in the food insecurity intervention came from program reports and published budgets from Nunavut and the Northwest Territories [46–49]. Lastly, the housing construction costs came from an Inuit Tapiriit Kanatami (ITK) report [67] outlining a housing strategy across the four Inuit homelands, and the annual maintenance costs reflected information from a Nunavut Housing Commission report [68].

**Table 4:** Risk factor reduction strategy costs (2018 $CAD)

Strategy	Components	Cost	Reference
combined Tobacco Reduction strategy^*^	(a) Total per-person cost of pharmacotherapy and counselling	$1749	[66, 69, 70]
	(b) Proportion of smokers who made a quit attempt	19.8%	[51]
	(c) Recommended per capita expenditure on mass media campaigns	$2.10	[65]
	*Prorated mean cost of pharmacotherapy, counselling and mass media per individual smoker (a* × *b) + c: added to all smokers*^a^	*$348*	
on the land HEALING CAMP	(a) Total per-person start-up cost of healing camps	$1599	[21]
	(b) Total per-person annual operating cost of healing camps	$37,444	[21]
	(c) Proportion of heavy drinking population participating in healing camp	7%	[21]
	*Prorated mean cost of healing camps per individual heavy drinker* *(a + b)*× *c: added to all heavy drinkers*	*$2695*	
combined food insecurity reduction strategy	(a) Total per-person cost for Niqinik Nuatsivik Nunavut Food Bank	$168	[46]
	(b) Total per-person cost for Canadian Prenatal Nutrition Program	$1481	[47]
	(c) Pregnant women as a proportion of the food insecure population	2.3%	[71]
	(d) Total per-person cost for Country Food Distribution Program	$412	[48]
	(e) Total per-person cost for community greenhouse project	$11	[49]
	*Prorated mean per person cost of reduction strategy per food insecure individual* *a + (b*×*c) + d + e : added to all food insecure individuals*	*$625*	
Housing construction	(a) Total per-person cost of building 426 new housing units	$151,494	[67, 68]
	(b) Total per-person cost of maintaining 426 housing unit annually	$2530	
	(c) Proportion of population in crowded housing moving to a new house	6%	[50]
	*Prorated mean per person cost per individual living in overcrowded housing* *(a + b)* ×*c: added to all individuals living in overcrowded housing*	*$9149*	

^a^Mass media costs also applied to non-smokers, as exposure to mass media campaigns is not exclusive to smokers

We did not assume that once community members became food secure, they would no longer need the food bank, greenhouse or country food program. As such, those who became food secure as a result of these programs continued to use them and incurred the associated cost. This is in contrast to pharmacotherapy, for example, where the population who quit smoking as a result of this program did not incur subsequent related smoking cessation costs.

#### TB-related health system costs

Costs associated with active TB and LTBI management and clinical care are summarized in Table 5. This includes TB-related diagnostic costs, treatment costs, and costs of adverse events associated with TB medications. The cost of treating active TB also includes transfers to Ottawa for patients requiring complex care, which occurs in approximately 4% of persons with active TB [35, 72]. TB-related health system costs were obtained from published sources from Nunavut, where available.

**Table 5:** TB-related health system costs in Nunavut (2018 $CAD)

Components	Cost	Reference
Tuberculin skin test	$19	[73]
9-month regimen of isoniazid	$186	[66]
Major adverse reaction to isoniazid	$15,269	[73]
Chest X-ray	$70	[74]
Three sputum samples analysis (when results are negative)	$30	[72]
Three sputum samples analysis (when results are positive; includes PCR probe)	$83	[72]
Spontaneous sputum production per sample	$3.60	[35]
Sputum induction for 3 samples	$99	[72]
Xpert® MTB/RIF test for one individual (1 sample)	$137	[35]
Standard 6-month active TB medication regimen (including Vitamin B6)	$655	[66]

*PCR* polymerase chain reaction

### Sensitivity and scenario analysis

Tornado diagrams were used to identify the most influential model parameters for each strategy. Additional sensitivity analyses focused on those parameters. In the case of the food insecurity reduction strategy, we started from an assumed odds ratio of 2.42 for becoming food secure, as described in Table 3 [53]. This was a much more conservative point estimate than in the study by Thompson and colleagues. However, we considered even more limited impact (odds ratio as low as 1.35) as well as up to 5.5, so as to remain symmetric on the logarithmic scale. Because of the marked uncertainty about the effect of programs in improving food security, we focused primarily on identifying thresholds where the food security intervention produced reductions in TB incidence similar to those afforded by programs targeting other risk factors. We also considered various combinations of the four strategies.

The Inuit Nunangat Housing Strategy estimates a current gap of 3500 housing units for Nunavut without accounting for population growth [67]. This strategy advocates for housing construction and improvement in housing conditions across Inuit Nunangat. As such, we considered additional scenarios where more than 426 houses were to be built in the overcrowding reduction strategy. We continued to assume that an average of 3.3 individuals would be accommodated in each house [50].

Probabilistic sensitivity analysis (PSA) was conducted by varying parameter estimates over their distributions (see Additional file 1 - Supplemental Table 5) and running 10,000 simulations to generate 95% uncertainty ranges (UR) for all model outputs.

## Results

### Base case

Compared to the status quo scenario with no new interventions directed at any of the four risk factors, the tobacco reduction strategy reduced TB incidence by 5.5% (95% UR: 2.6–11%) over 20 years. The reduction strategy for food insecurity reduced TB incidence by 1.8% (95% UR: 0.5–3.1%), the heavy drinking reduction strategy reduced it by 0.7% (95% UR: 0.4–0.9%), and the overcrowding reduction strategy at current scale reduced it by 1% (95% UR: 0.6–1.3%). In addition, the tobacco reduction strategy had the largest impact on TB-related deaths, reducing them by 13.4% (95% UR: 6.9–25.8%) over 20 years, and the largest effect on QALYs, which increased by 3.1 per 1000 persons (95% UR: 1.2–8.9) over 20 years relative to the status quo. Results are shown in Table 6.

**Table 6:** Projected costs and health outcomes per 1000 persons over 20 years

Outcomes per 1000 persons (95% UR)	Cost	TB Incidence	TB Deaths	QALYs
Status Quo	$1,199,205 ($765,946 → $1,883,900)	29.12 (19.06 → 43.21)	2.76 (1.32 → 6.25)	15,001.86 (14,968.20 → 15,014.70)
Tobacco Reduction Strategy	$1,353,274 ($946,333 → $1,994,712)	27.51 (18.13 → 40.40)	2.39 (1.15 → 5.35)	15,004.96 (14,975.35 → 15,016.31)
Heavy Drinking Reduction Strategy	$1,680,078 ($1,250,339 → $2,359,473)	28.92 (18.96 → 42.83)	2.73 (1.31 → 6.16)	15,002.17 (14,969.09 → 15,014.83)
Food Insecurity Reduction Strategy	$3,300,445 ($2,876,803 → $3,974,764)	28.58 (18.75 → 42.26)	2.71 (1.30 → 6.13)	15,002.32 (14,969.19 → 15,014.94)
Food Insecurity Reduction Strategy and Tobacco Reduction Strategy	$3,456,071 ($3,056,024 → $4,081,059)	27.02 (17.88 → 39.60)	2.35 (1.13 → 5.25)	15,005.36 (14,976.11 → 15,016.51)
Overcrowding Reduction Strategy	$8,282,029 ($7,854,241 → $8,955,843)	28.84 (18.93 → 42.73)	2.74 (1.31 → 6.19)	15,002.18 (14,968.91 → 15,014.86)
All Four Reduction Strategies in Combination	$11,021,792 ($10,630,433 → $11,635,312)	26.59 (17.67 → 38.87)	2.31 (1.12 → 5.12)	15,005.90 (14,977.39 → 15,016.78)

Incremental cost-effectiveness ratios (ICERs) comparing each strategy to the preceding strategy are shown in Table 7.

**Table 7:** Projected incremental costs and health outcomes per person over 20 years

Costs per person and ICERs, PRECEDING STRATEGY AS COMPARATOR	Incremental Cost (95% UR)	Incremental Cost per TB Case Averted (95% UR)	Incremental Cost per TB Death Averted (95% UR)	Incremental Cost per QALY Gained (95% UR)
Status Quo	–	–	–	–
Tobacco Reduction Strategy	$154 ($71 → $190)	$95,835 ($20,365 → $310,345)	$418,105 ($81,792 → $1,418,618)	$49,671 ($9152 → $157,357)
Heavy Drinking Reduction Strategy	$327 ($294 → $407)	Dominated^a^	Dominated	Dominated
Food Insecurity Reduction Strategy^b^	$1620 ($1602 → $1634)	Dominated	Dominated	Dominated
Food Insecurity Reduction Strategy and Tobacco Reduction Strategy^c^	$2103 ($2083 → $2116)	$4,274,725 ($1,994,219 → $15,347,313)	$52,609,991 ($17,372,467 → $217,605,661)	$5,275,987 ($2,126,824 → $17,906,886)
Overcrowding Reduction Strategy	$4826 ($4786 → $4911)	Dominated	Dominated	Dominated
All Four Reduction Strategies in Combination	$7566 ($7547 → $7578)	$17,647,965 ($9,772,005 → $37,319,412)	$160,913,981 ($60,384,312 → $472,384,673)	$13,924,008 ($5,977,290 → $32,253,031)

*ICER* Incremental Cost-Effectiveness Ratio

*Negative values indicate cost savings compared to the preceding strategy*

^a^*A strategy is dominated when it is both more costly and less effective than the preceding strategy*

^b^*The food insecurity reduction strategy is compared to the tobacco reduction strategy because the heavy drinking strategy is dominated. Because of this, the food insecurity strategy is dominated as well, due to extended dominance*

^c^*The combination of food insecurity reduction and smoking reduction is preferred to food security reduction alone, because of extended dominance. For that reason, incremental values are listed in comparison to the tobacco reduction strategy*

The tobacco reduction strategy was estimated to cost just under $50,000 per QALY gained. Strategies targeting each of the other risk factors alone were dominated, i.e. more expensive but less effective. Strategies involving combinations of interventions were much more expensive relative to health gains. Hence the combination of all four strategies had the highest anticipated reduction in TB incidence, but was also the most expensive, with an estimated incremental cost of nearly $14 million per QALY gained or nearly $18 million per TB case prevented. Combining the tobacco reduction and food insecurity reduction strategies yielded a similar reduction in TB incidence, but at a much lower cost. Specifically, its total cost was 68.6% lower (95% UR: 64.8–71.2%) and TB incidence only 1.6% higher (95% UR: 1.1–2.1%) when compared to the combination of all four strategies.

Table 8 shows the breakdown of costs, between the reduction strategies and the TB- related health system costs. While all strategies were associated with some savings on TB-related health system costs, the costs for each strategy itself consistently outweighed any savings.

**Table 8:** Projected health system and intervention costs per 1000 persons over 20 years

Outcomes per 1000 persons	Total Cost	Intervention Cost	TB-Related Health System Cost	TB-Related Health System Savings Compared to Status Quo^a^
Status Quo	$1,199,205	$0	$1,199,205	–
Tobacco Reduction Strategy	$1,353,274	$209,829	$1,143,444	$55,761
Heavy Drinking Reduction Strategy	$1,680,078	$487,941	$1,192,137	$7068
Food Insecurity Reduction Strategy	$3,300,445	$2,119,885	$1,180,560	$18,646
Overcrowding Reduction Strategy	$8,282,029	$7,092,984	$1,189,045	$10,161

^a^calculated as TB-related health system cost in status quo scenario – TB-related health system cost in intervention scenario

### Scenario and sensitivity analyses

Results of scenario analyses considering varying levels of housing construction are shown in Table 9. Meeting the 3500 units housing gap in Nunavut would further reduce TB incidence and related deaths by 6.9% over 20 years compared to the more limited scale of overcrowding reduction assumed in the base case scenario. Per-person costs and incremental cost-effectiveness ratios remain largely unchanged.

**Table 9:** Projected overcrowding reduction strategy outcomes per 1000 persons over 20 years

Number of Housing Units Built	Estimated Number of Individuals Accommodated	Cost	TB Incidence	TB Deaths	Quality Adjusted Life Years
426	1406	$8,282,029	28.84	2.74	15,002.18
1500	4950	$17,736,070	28.47	2.70	15,002.61
3500^a^	11,550	$57,714,176	26.84	2.55	15,004.47
7000	23,100^b^	$110,409,791	24.58	2.34	15,007.08
10,000	33,000	$152,537,566	22.60	2.15	15,009.31

^a^Number of housing units needed to close the housing gap according to the Inuit Nunangat Housing Strategy (INHS 2019)

^b^Approximately equal to the number of individuals living in overcrowded housing in Nunavut in 2018

Tornado diagrams (Additional file 1 - Supplemental Figs. 4–9) showed that predicted costs associated with each reduction strategy were most affected by the parameter representing the relative risk of progressing to active disease following infection for individuals living in overcrowded housing compared to individuals in non-crowded housing, as well as by the cost of hospitalisation.

When the food insecurity reduction strategies were assumed to reduce food insecurity by a factor of greater than 5.5 fold, they became more effective at reducing TB incidence than the tobacco reduction strategy. Despite becoming more effective for reducing incidence, the food insecurity reduction strategy was more expensive than the tobacco reduction strategy and was associated with higher relative TB mortality. This is because tobacco use is directly associated with increased TB case fatality, so that reduced tobacco use leads to lower case fatality. Further sensitivity analysis results are presented in Additional file 1 – section “Sensitivity analysis”.

## Discussion

Our analysis identified the tobacco reduction strategy as least costly and most effective in reducing TB morbidity and mortality. The strategies for heavy drinking reduction and food security were also beneficial, albeit at higher cost. The population-level impact of the housing intervention was constrained by limited reach and high cost at current scale. However, this intervention has potential to reduce TB morbidity and mortality substantially when scaled up and provides vital benefits, including improved mental health, education and food security [75, 76]. Similarly, the land-based healing camp reached only a fraction of the heavy-drinking population at current scale, but provides great benefit to those individuals. Both interventions may be scaled up to meet a community’s needs, but their costs would increase proportionally, so the per-person cost would remain similar.

Overall, the parameter representing the relative risk of progression to active disease among people in crowded homes compared to non-crowded homes was the most influential in driving costs and effectiveness. This may be because of the estimate’s magnitude, as well as the rise in overcrowding prevalence throughout the model’s analytic horizon.

The strategies for food security and reduction of heavy drinking both draw explicitly on the community’s culture, practices and strengths. However, these strategies also depend on materials from the South, e.g. construction and maintenance of community freezers, certain foods for the food banks, and building supplies for on-the-land healing camps. Transport of materials from the South has substantial cost implications, and is a driver for the high cost of these strategies, as well as the strategy to reduce overcrowding.

Although $50,000 per quality adjusted life year has been a frequent benchmark, Canadian guidelines do not recommend any specific threshold in assessing cost-effectiveness [77], while the United Kingdom and United States guidelines generally use thresholds of £50,000 GBP and $100,000 USD, respectively [77, 78]. Policy and program decisions should reflect not only cost-effectiveness estimates, but feasibility and cultural acceptability. Where possible, we based our risk factor reduction strategies on initiatives that already exist in remote settings to illustrate their feasibility.

It is important to consider some limitations of our study. We did not address other improvements in health related to mitigation of tobacco and alcohol use, food insecurity, and overcrowded housing. We did not consider how a change in prevalence of one TB risk factor might affect the prevalence of others, or whether the effect of certain risk factors was dose-dependent (i.e. how much an individual’s risk of TB would change when they used more tobacco). Where information on costs and effects of the reduction strategies selected was not available from Nunavut, we used information from other settings, which were not always specific to Inuit communities.

In some cases, our results reflect limited data with imprecise measures. Most importantly, a study conducted in 14 communities in Northern Manitoba found that persons in communities with a country food program were 20 times more likely to be food secure than in communities without such a program [53], but the confidence interval was extremely wide. We considered the lower bound of this interval as a conservative estimate of effect in our base case scenario due to the lack of other published information. As such, more data are needed with respect to the precise impact of such reduction strategies, especially because food insecurity is a prevalent issue.

Nonetheless, there is good reason to expect that investments that successfully mitigate these risk factors will reduce TB morbidity and mortality. In this sense, they will complement recent efforts to improve diagnostic capacity and prevention [79]. The relative isolation of many Indigenous communities highlights the role of preventive strategies that build on local resources. In Indigenous communities worldwide, building local capacity is key to addressing social and behavioural risk factors. Some key examples are initiatives that address tobacco use, heavy drinking and diabetes in Aboriginal peoples and Torres Strait Islanders in Australia [80–83].

Few studies from Indigenous settings have explored cost-effectiveness of community based interventions that target social and behavioural determinants of health. One study highlighted health gains associated with a community-based intervention to reduce diabetes and cardiovascular disease in a remote region of Western Australia [84]. Another Australian report suggested that price discounts to encourage healthy eating in remote Indigenous communities were in fact associated with poorer population health; instead, more holistic, culturally appropriate strategies were encouraged [85]. More generally, economic evaluations of health interventions in Indigenous settings have underscored the importance of leveraging local capacity and advocating community ownership [86]. These are also essential elements of TB prevention and care in non-Indigenous settings.

Economic analyses from non-Indigenous settings have emphasized the importance of addressing social and behavioural determinants of health. One report highlighted the cost-effectiveness of screening and brief interventions for excess alcohol use in European countries [87], while another underlined the cost-effectiveness of repeated tobacco cessation counselling in the United States [88]. Financial protection strategies to mitigate TB’s downstream social impacts have been identified as essential components of person-centred care [89]. Clearly, social and behavioural risk factors for TB will require concerted action if we are to meet key national and global TB reduction targets [2, 41, 90–93].

The history of TB in Canada’s North is tightly linked to colonization and colonial policies [3, 94, 95]. Hence it is all the more important to prioritize strategies led by Inuit community members, and based in Inuit culture and the land. The launch of the Canadian Tuberculosis Task Force to address TB [95] in Inuit communities has emphasized the significance of local partnerships to ensure such strategies are culturally appropriate, sustainable, and speak to the Inuit experience.

## Conclusion

Strategies that reduce commercial tobacco use will likely lead to modest improvement in tuberculosis morbidity and mortality in Canada’s North, and may be reasonably cost-effective. From the TB perspective, strategies to address food insecurity will have less impact at higher cost, although these bring other important health benefits. Similarly, strategies that address excess alcohol use and overcrowding will have a limited impact on TB-related outcomes at current scale. Their benefits will be more substantial with scale up. Strategies that address alcohol use and overcrowding will likely also have meaningful impact beyond TB, including improved mental health and educational attainment. These findings will also be relevant to other Indigenous and non-Indigenous communities and settings, where these risk factors and TB are co-prevalent.

## Supplementary Information

**Additional file 1:.** Supplemental methods and results. Contains additional details of dynamic model, decision analysis model, sensitivity analyses, dynamic model equations

## Data Availability

All models and relevant data inputs are available upon request to the corresponding author.

## Abbreviations

CDC: Centers for Disease Control and Prevention
CFDP: Country Food Distribution Program
CI: Confidence interval
CPNP: Canada Prenatal Nutrition Program
HR: Hazard ratio
ICER: Incremental cost-effectiveness ratio
INH: Isoniazid
ITK: Inuit Tapiriit Kanatami
LTBI: Latent tuberculosis infection
OR: Odds ratio
PCR: Polymerase chain reaction
PSA: Probabilistic sensitivity analysis
QALY: Quality adjusted life year
RAMQ: Régie de l’assurance maladie du Québec
RR: Relative risk
UR: Uncertainty range
