# Supplementary material for: Social and behavioral risk reduction strategies for tuberculosis prevention in Canadian Inuit communities: a cost-effectiveness analysis
Source: BMC Public Health. 2021 Feb 3;21:280. doi: 10.1186/s12889-021-10187-z (PMC7860224; doi:10.1186/s12889-021-10187-z)
Supplement: Supplementary file 1 — Additional file 1:. Supplemental methods and results. Contains additional details of dynamic model, decision analysis model, sensitivity analyses, dynamic model equations [file 12889_2021_10187_MOESM1_ESM.docx]

**Social and behavioural risk factor reduction strategies**

**for tuberculosis prevention in Inuit communities:**

**A cost-effectiveness analysis**

**Additional File 1**

**Table of Contents**

**METHODS** 1

Dynamic Model 1

Overview 1

Model Structure 1

Model calibration and validation 7

Phase 1 (Seeding to equilibrium) 7

Phase 2 (1984-1967) 8

Phase 3 (1967-2000) 8

Phase 4 (2000-2018) 8

TB risk factors over time 9

Calibration details 10

(1) Contact rate (b) 10

(2) Progression Rate (p) 10

(3) Immunity (im) 11

(4) Relapse after spontaneous recovery and relapse

after active treatment (v_SponRec and v_Active Tx) 11

Decision Analysis Model 12

Overview 12

Model structure 12

Data inputs 12

TB Pathogenetic and Epidemiologic parameters 12

TB Program parameters 13

Risk factor reduction strategies 13

(1) Tobacco reduction strategy 13

(2) Heavy drinking reduction strategy 13

(3) Food insecurity reduction strategy 14

(4) Overcrowded housing reduction strategy 14

Sensitivity analysis 18

**RESULTS** 20

**SLIR MODEL EQUATIONS** 28

Strata and Corresponding Risk Factors 28

TB Health States 28

Initial Conditions 29

Difference Equations 30

Infection Multipliers 31

Mortality Multipliers 31

Progression Multipliers 31

Parameter Values 32

Pathogenetic Parameters (Stratum 16) 32

Pathogenetic Parameters (all other strata) 32

Other Parameters (Common to all strata) 32

Transition Rates 32

**REFERENCES** 34

Methods

Dynamic Model

Overview

In order to capture the complexity of the history of tuberculosis (TB) in the Eastern Arctic, we created a susceptible-latent-infectious-recovered (SLIR) TB transmission model. This model was created to describe key historical TB-related events such as the pre-antibiotic era, the shift in living conditions, the introduction of antibiotics, and recent prevention and care measures. We informed model parameters using published literature, and calibrated unknown parameters against observed data. By simulating the spread of TB in this region, we were able to predict key measures such as the annual risk of infection (ARI), the incidence of active TB, and TB-related mortality. These measures, along with certain model parameters and population distributions within TB-related health states were used as initial conditions in a decision analysis model that analyzed the cost-effectiveness of TB risk factor reduction strategies over a 20-year horizon.

Model structure

Difference equations governed the transition of a hypothetical cohort through various TB-related health states, representing the spread of this disease from one community member to another. At the same time, we considered social and behavioural risk factors for TB; trends in commercial tobacco use, heavy drinking, food insecurity, and overcrowded housing prevalence were incorporated into the model by stratifying individuals based on their risk factor combination (see Supplemental Figure 1). For example, if an individual had all four risk factors, they were placed in a unique stratum. In this stratum, they transitioned between TB-related health states with a stratum specific probability. If they initiated or no longer had a certain risk factor (e.g. if they stopped smoking, if their housing became overcrowded), they transitioned to another stratum. The rate at which they transitioned between strata depended on the prevalence of the four TB risk factors over time in the region (see Supplemental Table 3).

This model also incorporated reported estimates of effect that each TB risk factor had on rates of infection, progression, reactivation, and death wherever appropriate. For example, heavy drinking, defined as consuming more than 40 grams of alcohol per day and/or having an alcohol use disorder, was estimated to confer a fourfold increase in progression from latent infection to active disease [^[[1]](#endnote-1)^]. Consequently, the rapid progression parameter was multiplied by this estimate of effect in each of the heavy drinking strata, and four times the number of individuals who were not heavy drinkers progressed to the active disease state.

**Supplemental Figure 1. Stratification of hypothetical cohort in dynamic model based on social risk factor combination**

Model parameters directed the transition between health states and risk factor strata. These health states are depicted in Supplemental Figures 2 and 3, and model parameters are described in Supplemental Table 1.

A detailed description of the rationale behind each TB-related health state is available in the Supplementary Appendix of N’Diaye et al. 2019 [^[[2]](#endnote-2)^].

**Supplemental Table 1. Dynamic model parameters**

|  |  | **PARAMETER VALUE*** | | |
| --- | --- | --- | --- | --- |
| **PARAMETER** | **DESCRIPTION** | **1948** | **2000** | **2018** |
| **ARI** | annual risk of infection; calculated using b | 25.24 | 0.18 | 0.73 |
| **p** | rate of primary progression from LTBI to active TB^†^ | 0.0091^#^ | 0.000035^#^ | 0.0018^#^ |
| **v** | annual reactivation rate from long standing LTBI to active TB^‡^ | 0.001^#^ | 0.0005^#^ | 0.0005^#^ |
| **t** | transition rate from latent fast to latent slow^‡^ | 0.25 | 0.25 | 0.25 |
| **z** | death rate of untreated TB^‡^ | 0.065^#^ | 0.074^#^ | 0.077^#^ |
| **d** | natural cure rate^‡^ | 0.25 | 0.25 | 0.25 |
| **tx_rate** | active TB treatment rate^‡^ | 1 | 1 | 1 |
| **d_rate** | background mortality rate^‡^ | 0.023 | 0.016 | 0.019 |
| **prot_ltbi** | sensitivity and efficacy of LTBI treatment^‡^ | 0.4833 | 0.4833 | 0.4833 |
| **im** | immunity conferred by primary infection^†^ | 0.55 | 0.55 | 0.55 |
| **cd** | diagnosis rate for active TB^‡^ | 0 | 0.9 | 0.9 |
| **cd_latent** | diagnosis rate for latent disease^‡^ | 0 | 0.03 | 0.03 |
| **txs** | probability of active treatment completion and success^‡^ | 0.97 | 0.97 | 0.97 |
| **zd** | death rate under active TB treatment^‡^ | 0.014^#^ | 0.016^#^ | 0.017^#^ |
| **b** | beta, contact rate^†^ | 0.925 | 0.30 | 0.87 |
| **v_SponRec** | reactivation rate after spontaneous recovery | 0.025^‡#^ | 0.0048^†#^ | 0.016^†#^ |
| **v_ActiveTx** | reactivation rate after active treatment | 0.015^‡#^ | 0.0029^†#^ | 0.014^†#^ |
|  | Model entry and exit flows of individuals |  |  |  |
|  | Flows between compartments |  |  |  |
| ***** For parameter values that change over time, see SLIR model equations at the end of this Additional File 1 for exact trends  ^#^ These values applied to the stratum of individuals with no specific TB risk factors; parameters for all other strata were calculated by multiplying these values by their respective estimates of effect (see SLIR equations at the end of this document for details)  ^†^ Calibrated against observed ARI and incidence rate using the dynamic model; see Supplemental Table 2  ^‡^ Values based on those from literature [^[[3]](#endnote-3)^,^[[4]](#endnote-4)^,^[[5]](#endnote-5)^,6,7,8] | | | | |

**Supplemental Figure 2. Equilibrium model (Pre-1948)**

**
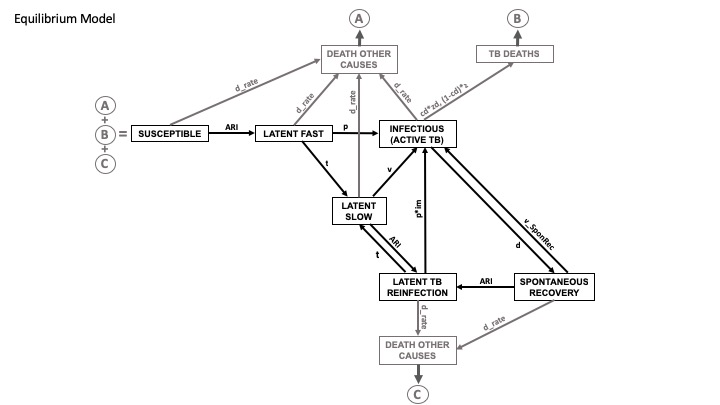
**

**Supplemental Figure 3. Model with diagnosis and treatment of latent TB infection and active TB (Post-1948)**

**
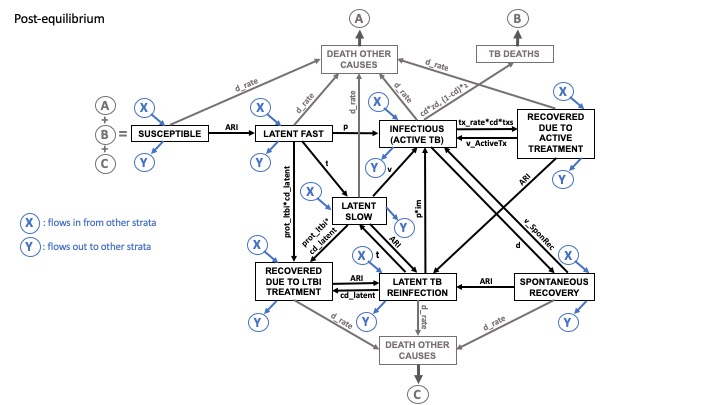
**

Model calibration and validation

The dynamic model was simulated in four phases, described in detail below. Each phase marked a key event in history (for which we had reliable data on TB epidemiology to calibrate the model), such as the advent of antibiotic use, or changes in living conditions. To capture these changes in the model, key parameters were calibrated. At each stage, the model’s TB incidence rate and ARI were validated against observed measures, as seen in Supplemental Table 2.

**Supplement Table 2. Observed TB incidence rate and Annual Risk of Infection used for calibration**

| Time Point in the Model | Corresponding Year | Observed Annual Risk of Infection (ARI) | Observed Incidence Rate (per 100,000 person-years) |
| --- | --- | --- | --- |
| 0 | 1948 | 25 | 2900 |
| 17 | 1965 |  | 1490 |
| 23 | 1971 | 3.5 |  |
| 26 | 1974 | 1.75 |  |
| 52 | 2000 |  | 172.9 |
| 53 | 2001 |  | 139.5 |
| 54 | 2002 |  | 93.7 |
| 55 | 2003 |  | 23.9 |
| 56 | 2004 | 0.33 | 107.2 |
| 57 | 2005 |  | 148.4 |
| 58 | 2006 |  | 155.8 |
| 59 | 2007 |  | 98.7 |
| 60 | 2008 |  | 185 |
| 61 | 2009 |  | 168.7 |
| 62 | 2010 |  | 299.8 |
| 63 | 2011 |  | 216.4 |
| 64 | 2012 |  | 230.5 |
| 65 | 2013 |  | 143.3 |
| 66 | 2014 |  | 229.6 |
| 67 | 2015 |  | 119.2 |
| 68 | 2016 |  | 142.9 |

Phase 1 (Seeding to Equilibrium)

A hypothetical cohort was spread across 16 strata according to the initial prevalence of each TB risk factor, which we estimated based on observed data (shown in Supplemental Table 3). One infectious individual was introduced to a completely susceptible population, and the model was run until each health state’s distribution was stable (i.e. until the model reached equilibrium). This simulated the natural history of TB prior to any intervention, such as treatment, in order to evaluate key infection parameters prior to the initiation of public health measures. That’s why the model’s structure in Supplemental Figure 2 looks simpler than in Supplemental Figure 3 – the two recovery-following-treatment states weren’t populated by any cohort member because diagnosis and treatment for LTBI and active TB didn’t exist in this phase. In addition, three parameters were calibrated against observed ARI and TB incidence: the contact rate (b), the progression rate (p) and immunity conferred by previous infection (im). Calibration is described in more detail following Supplemental Table 3. Upon reaching equilibrium, these parameter values and the corresponding cohort distribution across the TB-related health states in each of the 16 strata were used as initial conditions for Phase 2, which started in 1948.

Phase 2 (1984-1967)

This period saw changes in living conditions and a documented decline in the contact rate [^[[6]](#endnote-6)^,^[[7]](#endnote-7)^,^[[8]](#endnote-8)^,^[[9]](#endnote-9)^]. Subsequently, TB incidence began to decline; this was not due to TB program improvement because no new interventions were implemented at this time. In order to reflect this trend, we introduced a decline in the contact rate (b) and progression rate (p) parameters. Again, we made sure to validate the model’s ARI and TB incidence rate with what was observed.

Phase 3 (1967-2000)

1967 marked the year when diagnosis and treatment of LTBI and active TB were widely introduced to the region. This is when individuals began populating the two recovery-following-treatment compartments that were previously empty. Corresponding transition rates were introduced, along with the assumption that diagnosis and treatment rates increased linearly and eventually plateaued. During this period, the contact rate and progression rate continued to drop in order to capture the improvements in TB management. In addition to these two parameters, we introduced a decline in the relapse rates following active treatment (v_ActiveTx) and spontaneous recovery (v_SponRec); we assumed that following the initiation of TB diagnosis and treatment, relapse rates would begin to fall.

Phase 4 (2000-2018)

In recent decades, the incidence of TB has been increasing in Arctic regions [^[[10]](#endnote-10)^,^[[11]](#endnote-11)^]. To simulate this upturn, the four parameters (b, p, v_ActiveTx and v_SponRec) that were previously declining, began to increase. None of the four alone were sufficient in replicating the increasing incidence, and neither was the combination of b and p. As such, we introduced a steady rise in the four parameters and validated the model’s incidence rate and ARI against what was observed. Similar to the first three phases, we justified using these four parameters on the epidemiologic basis of declining general health that hampered spontaneous recovery, increased the risk of relapse and progression, and increased the contact rate.

TB risk factors over time

The prevalence of the four social and behavioural TB risk factors over time dictated how members of the cohort moved between strata. Prevalence data from recent decades was generally available and was used to extrapolate trends where data was missing. For heavy drinking prevalence, for example, the trend line was extrapolated to previous decades and resulted in a steady linear increase from 13.5% to 18.1% from 1948 to 2018. Data on commercial alcohol product sales was available from early decades and showed a similar steady increase over time, so we used that to cross check our estimates [^[[12]](#endnote-12)^,^[[13]](#endnote-13)^]. Similarly, for food insecurity prevalence, we interpolated recent trends between available data points and checked that they matched up with trends observed in fruit and vegetable consumption [^[[14]](#endnote-14)^]. Supplemental Table 3 outlines the observed data points for each TB risk factor.

**Supplemental Table 3. Observed prevalence of social and behavioural risk factors for TB**

| Time Point in the Model | Corresponding Year | Smoking prevalence | heavy drinking prevalence | food insecurity prevalence | overcrowded housing prevalence |
| --- | --- | --- | --- | --- | --- |
| 48 | 1996 |  | 12.6% |  | 61.0% |
| 49 | 1997 |  |  |  |  |
| 50 | 1998 |  | 12.0% |  |  |
| 51 | 1999 |  |  |  |  |
| 52 | 2000 |  | 17.9% | 56.0% |  |
| 53 | 2001 | 56.0% |  |  | 53.0% |
| 54 | 2002 |  |  |  |  |
| 55 | 2003 | 64.8% | 13.2% |  |  |
| 56 | 2004 |  |  |  |  |
| 57 | 2005 | 53.1% | 14.6% |  |  |
| 58 | 2006 |  |  |  | 39.0% |
| 59 | 2007 | 59.0% | 12.7% | 68.8% |  |
| 60 | 2008 | 54.2% | 16.2% |  |  |
| 61 | 2009 | 61.3% | 12.5% |  |  |
| 62 | 2010 | 54.4% | 8.7% |  |  |
| 63 | 2011 | 59.7% | 17.6% |  | 39.2% |
| 64 | 2012 | 54.3% | 14.9% | 56.0% |  |
| 65 | 2013 | 59.0% | 16.4% |  |  |
| 66 | 2014 | 62.0% | 14.4% | 46.8% |  |
| 67 | 2015 |  |  | 50.8% |  |
| 68 | 2016 |  |  |  | 56.4% |
|  |  | SMOKING  DATA: [^[[15]](#endnote-15)^,^[[16]](#endnote-16)^] | HEAVY DRINKING DATA:[16] | FOOD INSECURITY DATA:[^[[17]](#endnote-17)^,^[[18]](#endnote-18)^,^[[19]](#endnote-19)^,^[[20]](#endnote-20)^] | OVERCROWDED HOUSING DATA: [^[[21]](#endnote-21)^,^[[22]](#endnote-22)^,^[[23]](#endnote-23)^] |

Calibration details

The model was calibrated against two observed outcomes: ARI and TB incidence rate (see Supplemental Table 2 for observed data). During Phase 1, the progression rate, contact rate, and immunity parameters were calibrated. During Phase 2, the progression rate and contact rate parameters were calibrated. Lastly, during Phase 3 and 4, the progression rate, contact rate, relapse after active treatment, and relapse after spontaneous recovery parameters were calibrated.

The first step in this calibration process was to linearly approximate a trend line between the observed values for both incidence rate and ARI, and interpolate and values between observed data points. Data sets were then imported into Berkeley Madonna (Version 8.3.23.0, University of California, 2015). The program’s “Curve Fit” calibration tool was used to fit model parameters, based on a Root Mean Squared (RMS) deviation method. The program’s “slider” tool was then used to further refine parameters so that the modelled ARI and incidence rate could be brought closer to their observed values, while making sure that parameters stayed within reasonable bounds, which were informed by literature. This procedure was performed for both the equilibrium model (pre-1948) and the post-equilibrium model (1948-2018). We’ve provided more detail below specific to each parameter that was calibrated:

1. **Contact Rate (b)**

The contact rate parameter began at a value of 0.925 in 1948. It declined quite rapidly until 1967, at which point it continued to decline, but at a slower rate until it reached its trough at a value of 0.3. The rapid decline reflected the changes in living conditions, as well as the documented drop in the rate of contact between individuals [7]. In the year 2000, however, this parameter started increasing to reflect the rising TB incidence. Although this parameter is not specific to the stratum with no TB risk factors, it was multiplied by estimates of effect on infection for each TB risk factor in calculating each stratum’s ARI (this is why the beta parameter’s value is so low). The 16 ARI estimates were then used to estimate an average ARI, which was matched with observed data.

1. **Progression Rate (p)**

The progression parameter began at a value of 0.0091 in 1948. It experienced a steady decline until 1967, at which point it began to decline exponentially, reaching a value of 0.000035. The exponential decline reflected the advent of diagnosis and treatment in 1967, upon which we assumed the rate of progression to active disease would decline as well. Similar to the contact rate parameter, the progression parameter began increasing following the year 2000 to reflect the rising TB incidence. In each stratum, this progression rate was multiplied by the appropriate estimates of effect based on TB risk factor combination (which is why the value listed in this paragraph is so low).

1. **Immunity (im)**

This parameter reflected the immunity rate conferred by primary infection, and did not change over time like the other two. It was, however, calibrated in the first phase to be equal to 0.55, and remained as such for the duration of the simulation.

1. **Relapse after spontaneous recovery and relapse after active treatment**

**(v_SponRec and v_ActiveTx)**

These two relapse parameters had values from literature in the first two phases and were calibrated to change starting in the third phase. As diagnosis and treatment were introduced, the two relapse parameters began to decline to reach values of 0.0048 (v_SponRec) and 0.0029 (v_ActiveTx). Similar to the contact rate and progression rate parameters, in the year 2000, the two relapse parameters started increasing. The reason for these changes was to bring the modelled ARI and incidence rate closer to what was observed. Changing only the contact rate and progression rate wasn’t sufficient in doing so. Consequently, these two parameters were calibrated as well.

There were three additional parameters that changed over time, but weren’t calibrated. Rather, their trends were based on estimates informed by literature. The parameters in question are the background mortality rate (d_rate), reactivation (v), and two case detection parameters (cd and cd_latent). Details of how their trends were estimated can be found in the Supplemental Appendix of N’Diaye et al. 2019 [2]. Their trendlines, however, can be found in the SLIR model equations at the end of this Additional File 1.

**Decision Analysis Model**

Overview

Once the dynamic model reached the end of its simulation, we used its population distributions and key parameters to act as initial conditions for the decision analysis model. For parameters that were not informed by the dynamic model, we obtained values from published literature, using Inuit specific data wherever possible. This model was then used to simulate various TB risk factor reduction strategies over 20 years in comparison to a “status quo” scenario where no new interventions targeting social and behavioural risk factors were put in place.

Model structure

Health states in the decision analysis model were the same as those in the dynamic model. Namely, there were susceptible, latent, active and recovered states, as depicted in Figure 2 in the main article. There were two additional states relating to mortality: death due to TB, and death from other causes. Each of these states was stratified by TB risk factor combination. At the end of every Markov cycle, which was one year in length, individuals would have the opportunity to switch strata based on annual cessation or initiation probabilities for each TB risk factor. This process is described by Figure 1 in the main article. After running 20 Markov cycles, outcomes were assessed, including TB incidence, TB-related mortality, quality adjusted life years (QALYs) and costs from the government perspective.

### Data inputs

TB Pathogenetic and Epidemiologic parameters

The parameters that came from the dynamic model were: the initial distribution of the population across the various health states (stratified by TB risk factor combination), the initial annual risk of infection (in 2018) for those with no specific TB risk factors, the probability of progression to active TB during the first 2 years after newly acquired infection for those with no specific TB risk factors, the probability of relapse following active treatment, and the probability of relapse following spontaneous recovery. Other parameters came from the literature, and are listed in detail in Supplemental Table 6.

TB Program parameters

Current guidelines in Nunavut recommend using a rapid automated PCR test (Xpert^®^ MTB/RIF) for the diagnosis of active TB [^[[24]](#endnote-24)^] and TB treatment should occur in an outpatient setting, with occasional transfers (4%) south to Ottawa for patients with complications and/or complex medical situations. In our models, we assumed that 90% of persons with active TB were diagnosed and treated, with 100% treatment completion [^[[25]](#endnote-25)^]. For latent TB infection (LTBI), we assumed that 25% of persons infected would be diagnosed and of those diagnosed, 47% started on treatment for LTBI [^[[26]](#endnote-26)^]. For those who start treatment, the probability of LTBI treatment completion was 75.6% based on Nunavut program data [26].

Risk factor reduction strategies

The strategies chosen to incorporate into the model were a combination of pharmacotherapy, counselling, taxation and mass media campaigns for commercial tobacco use, an on-the-land healing camp for heavy drinking, a community food distribution program for food insecurity, and social housing construction for overcrowding. Each strategy’s components are detailed below. Supplemental Table 4 describes how each strategy’s impact was integrated into the model. Supplemental Table 5 elaborates on the components of each strategy’s costs.

1. **Tobacco reduction strategy**

The tobacco use study considers several cessation strategies separately, and in combination [2]. Namely, the combined strategy consists of pharmacotherapy coupled with counselling, mass media campaigns and tobacco taxation. Pharmacotherapy entails 12 weeks of nicotine replacement therapy (NRT) combined with varenicline medication. Group and individual counselling is offered in tandem to enhance the odds of quitting [^[[27]](#endnote-27)^,^[[28]](#endnote-28)^,^[[29]](#endnote-29)^]. At the same time, there exist community-wide tobacco cessation media campaigns designed for Indigenous audiences [^[[30]](#endnote-30)^], as well as increased taxes applied to commercial tobacco products [^[[31]](#endnote-31)^,^[[32]](#endnote-32)^,^[[33]](#endnote-33)^,^[[34]](#endnote-34)^]. We used the combined strategy in this model because it was identified as the most effective approach to reducing tobacco use in the previous study [2]. As in that study, all aforementioned strategies were once-off (occurring in this scenario’s first year of simulation, 2018) [2].

1. **Heavy drinking reduction strategy**

The heavy drinking reduction strategy is centered on a land-based healing camp [^[[35]](#endnote-35)^]. Such a program currently operates out of the Cambridge Bay Wellness Centre, and is set to expand to two other regions in Nunavut [^[[36]](#endnote-36)^]. The strategy assumes a 28-day inpatient camp offered 3 to 4 times per year. Activities during the day focus on clinical recovery, and programming in the evening focuses on cultural activities (e.g. seal and goose hunting, traditional games, egging). Programs are offered in Inuinnaqtun and incorporate Inuit values with the involvement of Elders [36]. For our analysis, we assumed the program was scaled up in the three regions (Kitikmeot, Kivalliq and Qikiqtaaluk) by a factor of 5, so as to achieve sufficient population coverage. Costs were scaled up proportionally, based on published budgets [36].

1. **Food insecurity reduction strategy**

We considered a comprehensive approach to reducing food insecurity that incorporated initiatives based in Nunavut and the Northwest Territories. These initiatives fit within themes identified by a scoping review from the Inuvialuit region of the Northwest Territories [^[[37]](#endnote-37)^]. Specifically, we considered as prototype programs the Niqinik Nuatsivik Nunavut Food Bank, the First Nations and Inuit component of the Canada Prenatal Nutrition Program (CPNP), Nunavut’s Country Food Distribution Program (CFDP), and a community greenhouse project in Hay River, Northwest Territories in combination [46,47,48,49]. Based on the available information, costs were adjusted to reflect full coverage of the food-insecure population (or in the CPNP’s case, all food-insecure pregnant women).

1. **Overcrowded housing reduction strategy**

In 2014-2015, 216 housing units were built in 8 communities in Nunavik, and 210 housing units were built in 12 communities in Nunavut [^[[38]](#endnote-38)^]. These housing units were a combination of one-, two- and four-bedroom houses and apartments to accommodate a range of household sizes. Regions where the housing shortage was most severe were given priority in supplying additional units, and tenants were allocated accordingly with the help of local housing committees. A study reported a substantial decline in overcrowding after participants moved to a new house [38]. We subsequently used this 426-unit construction scenario as the overcrowded housing reduction strategy in our model. We assumed that the construction was once-off and that each new unit required yearly maintenance until the end of the simulation.

**Supplemental Table 4. Calculation of anticipated impact of reduction strategies on TB risk factor prevalence**

| CHANGE IN PREVALENCE OF TB RISK FACTOR WITHOUT REDUCTION STRATEGY | ESTIMATE OF EFFECT OF REDUCTION STRATEGY | REDUCTION STRATEGY’S ANNUAL REACH | DURATION OF REDUCTION STRATEGY AND ITS EFFECT | CALCULATION OF CHANGE IN PREVALENCE OF TB RISK FACTOR WITH REDUCTION STRATEGY |
| --- | --- | --- | --- | --- |
| SMOKING | | | | |
| Decrease in number of smokers by 0.013% per year | Pharmacotherapy, counselling and mass media: Relative Risk = 2.36 (95% confidence interval 1.01-5.50) [30]  Taxation: 7% reduction in smoking prevalence within three years and increases over time to 14% [31,^[[39]](#endnote-39)^] | Pharmacotherapy, counselling and taxation: 100% of the smoking population  Mass media: 100% of the general population | All strategies are once-off* Pharmacotherapy, counselling and mass media’s effect lasts 3 years. Taxation’s effect lasts 10 years. | First 3 years: 2.36 $\times$ ((7% $\div$3yrs) + 0.013%) = 5.54% decrease in number of smokers per year  Next 7 years: (7% $\div$7yrs) + 0.013% = 1.01% decrease in number of smokers per year  Next 10 years: back to background decrease in number of smokers by 0.013% per year |
| HEAVY DRINKING | | | | |
| Increase in number of heavy drinkers by 0.07% per year | 28.3% absolute reduction in use of alcohol and drugs after treatment session [^[[40]](#endnote-40)^] | ~7% of the heavy drinking population | Treatment cycle is once-off. As such, the effect occurs in the first year of the simulation, and doesn’t last beyond that. | First year: (28.3% $\times$ 7%) – 0.07% = 1.91% decrease in the number of heavy drinkers  Next 19 years: back to background increase in the number of heavy drinkers by 0.07% per year |
| FOOD INSECURITY | | | | |
| Decrease in number of persons living with food insecurity by 0.36% per year | Presence of a country food program increases the likelihood of being food secure; Odds Ratio = 20.64 (95% confidence interval 2.42-176.08)** [^[[41]](#endnote-41)^]. | Food bank, country food, greenhouse: 100% of the food insecure population  Canada Prenatal Nutrition Program: only food-insecure pregnant women (~ 2.3% of the food insecure population) | Each of these programs occurs yearly (except for the Canadian Prenatal Nutrition Program, which is once-off).  As such, the estimate of effect is applied to the food-insecure cohort each year. | Each year for 20 years: 2.42 $\times$ 0.36% = 0.87% decrease in the number of food-insecure individuals |
| OVERCROWDING | | | | |
| Increase in persons living in overcrowded housing by 2.27%-5.76% per year | 46% absolute reduction in overcrowding (65.5% at baseline vs. 19.5% at follow-up) [38]. | ~6% of the population living in overcrowded housing | Housing construction is once-off. As such, the effect occurs in the first year of the simulation, and doesn’t last beyond that. | First year: (46% $\times$ 6%) – 2.27% = 0.49% decrease in the number of persons living in overcrowded housing  Next 9 years: back to background increase of 2.27%-5.76% per year in the number of persons living in overcrowded housing  Next 10 years: no change in number of persons living in overcrowded housing (prevalence of overcrowded housing reaches 100% after 10 years of the simulation) |

*****Once-off means that the strategy only occurred within the first year of the simulation (2018)

****** Because of the uncertainty around the point estimate and its magnitude, we have used the lower bound of the 95% confidence interval as a conservative measure of impact: 2.42 with a 95% confidence interval that is roughly symmetric around it on the logarithmic scale: 1.35 – 5.5

| Supplemental Table 5. TB risk factor reduction strategy component costs (2018 $CAD) | | |
| --- | --- | --- |
| Components | Cost | Reference |
| COMBINED SMOKING CESSATION STRATEGY | | |
| (a) total cost of increased taxation (no cost to the government; costs only incurred by individual) | $0 |  |
| (b) unit cost of NRT chewing gum | $0.26 | ^[[42]](#endnote-42)^ |
| (c) number of doses of NRT chewing gum | 1008 | ^[[43]](#endnote-43)^ |
| (d) unit cost of nicotine patch | $2.75 | 42 |
| (e) number of doses of nicotine patch | 84 | 43 |
| (f) unit cost of Varenicline 0.5mg pill | $1.76 | 42 |
| (g) number of doses of Varenicline | 317 | 2 |
| (h) cost of treatment with NRT, nicotine patch and Varencline (a $\boldsymbol{\times}$ b) + (c $\boldsymbol{\times}$ d) + (e $\boldsymbol{\times}$ f) | $1,048.73 |  |
| (i) number of counselling cessions | 12 | 27 |
| (j) duration of each counselling session | 42 min | 27 |
| (k) hourly nurse wage | $83.39 | ^[[44]](#endnote-44)^ |
| (l) cost for complete counselling sessions (i $\boldsymbol{\times}$ j $\boldsymbol{\times}$k) | $700.46 |  |
| (m) total per-person cost of pharmacotherapy and counselling (h + l) | $1,749.19 |  |
| (n) proportion of smokers who made a quit attempt | 19.8% | 2 |
| (o) recommended per capita expenditure on mass media campaigns | $2.10 | ^[[45]](#endnote-45)^ |
| *Prorated mean cost of pharmacotherapy, counselling and mass media per smoker*  *(m* $\boldsymbol{\times}$ *n) + o: added to all smokers*^#^ | ***$348.44*** |  |
| ON THE LAND HEALING CAMP | | |
| (a) start-up costs for Qikiqtaaluk region | $297,481 | 36 |
| (b) start-up costs for Kivalliq region | $306,856 | 36 |
| (c) start-up costs for Kitikmeot region | $162,943 | 36 |
| (d) annual operating costs for Qikiqtaaluk region | $1,033,647 | 36 |
| (e) annual operating costs for Kivalliq region | $1,052,045 | 36 |
| (f) annual operating costs for Kitikmeot region | $1,508,931 | 36 |
| (g) number of people that can participate in healing camps | 96 | 36 |
| (h) scale up factor | 5 | Assumption |
| (i) total per-person cost of healing camps (a + b + c + (h $\boldsymbol{\times}$ (d + e + f))) $\boldsymbol{\div}$ (h $\boldsymbol{\times}$ g) | $39,042.49 |  |
| (j) estimated number of heavy drinking individuals in population | 6954 | 16 |
| (k) proportion of heavy drinking population in healing camps (g $\boldsymbol{\times}$ h) $\boldsymbol{\div}$ j | 7% |  |
| *Prorated mean cost of healing camps per heavy drinker*  *(i* $\boldsymbol{\times}$ *k) : added to all heavy drinkers* | ***$2694.91*** |  |
| COMBINED FOOD INSECURITY REDUCTION STRATEGY | | |
| (a) total cost of Niqinik Nuatsivik Nunavut food bank | $84,154.21 | 46 |
| (b) Niqinik Nuatsivik Nunavut food bank coverage (number of people) | 500 | ^[[46]](#endnote-46)^ |
| (c) total per-person cost for Niqinik Nuatsivik Nunavut food bank (a ÷ b) | $168.30 |  |
| (d) total cost of CPNP | $179,222.52 | ^[[47]](#endnote-47)^ |
| (e) CPNP coverage (number of pregnant women) | 121 | 47 |
| (f) total per-person cost of CPNP (d ÷ e) | $1,481.17 |  |
| (g) total cost of CFDP: freezer construction | $414,420.17 | ^[[48]](#endnote-48)^ |
| (h) total cost of CFDP: freezer repair | $725,652.60 | 48 |
| (i) total cost of CFDP: payments to hunters for harvesting | $15,028.79 | 48 |
| (j) number of individuals that benefitted from a new freezer | 14,651 | 17,18,19,20,23 |
| (k) number of individuals that benefitted from freezer repairs | 1,982 | 17,18,19,20,23 |
| (l) number of individuals that benefitted from harvesting | 835 | 17,18,19,20,23 |
| (m) total per-person cost of CFDP (g ÷ j) + (h ÷ k) + (i ÷ l) | $412.41 |  |
| (n) total cost of community greenhouse in Hay River | $42,506 | ^[[49]](#endnote-49)^ |
| (o) community greenhouse coverage (number of people) | 3824 | 17,18,19,20,23 |
| (p) total per-person cost of community greenhouse (n ÷ o) | $11.12 |  |
| (q) number of food-insecure individuals in the model | 21,502 | 17,18,19,20,23 |
| (r) number of food-insecure pregnant women in the model | 488 | 17,18,19,20,23 |
| *Prorated mean cost of combined food insecurity reduction strategy*  *(c + (f × (r ÷ q)) + m + p): added to all food insecure individuals* | ***$625.44*** |  |
| HOUSING CONSTRUCTION | | |
| (a) average cost of new housing unit construction | $500,000 | ^[[50]](#endnote-50)^  ^[[51]](#endnote-51)^ |
| (b) average cost of maintaining each housing unit annually | $8354.25 |  |
| (c) number of housing units built | 426 | 38 |
| (d) average number of individuals per house | 3.3 | 38 |
| (e) estimated number of individuals moving to a new house (c $\boldsymbol{\times}$ d) | 1406 |  |
| (f) total per-person cost of new housing unit construction (a $\boldsymbol{\times}$ c) $\boldsymbol{\div}$ e | $151,494 |  |
| (g) total per-person cost of maintaining each housing unit annually (b $\boldsymbol{\times}$ c) $\boldsymbol{\div}$ e | $2,530.44 |  |
| (h) estimated number of individuals living in overcrowded housing | 23,671 | 21,22,23 |
| (i) proportion of population in crowded housing moving to a new house (e $\boldsymbol{\div}$ h) | 6% |  |
| *Prorated mean cost of housing construction per individual living in overcrowded housing ((f + g)* $\boldsymbol{\times}$*i): added to all individuals living in overcrowded housing* | ***$9,148.70*** |  |

Sensitivity analysis

Supplemental Table 6 describes the decision analysis model parameters. Their distributions and ranges were used in sensitivity analysis to investigate which parameters have the most prominent impact on the model’s outcomes.

| Supplemental Table 6. Sensitivity analysis – Distributions of input parameters | | | | |
| --- | --- | --- | --- | --- |
| Description | Distribution | Point Estimate | Range | Reference |
| Cost of 7 outpatient clinic visits during Isoniazid treatment^†^ | Triangular | $622.78 | [$467.08 - $778.47] | ^[[52]](#endnote-52)^,^[[53]](#endnote-53)^ |
| Cost of major adverse reaction to Isoniazid^†^ | Triangular | $15,907.66 | [$11,930.74 – 19,884.57] | 52 |
| Cost of a chest X-ray | Triangular | $72.45 | [$54.33 - $90.56] | ^[[54]](#endnote-54)^ |
| Cost of a day of hospitalization | Triangular | $2,646.29 | [$1,984.72 - $3,307.87] | 24 |
| Hourly wage for a doctor only for non-remote community^†^ | Triangular | $70.85 | [$53.14 - $88.56] | 24 |
| Cost of initial TB assessment in a remote community: nurse fee^†^ | Triangular | $27.73 | [$20.80 - $34.67] | 24 |
| Cost of drug for DOT | Triangular | $655.09 | [$491.32 - $818.87] | 42 |
| Cost of Isoniazid, 9 months’ supply^†^ | Triangular | $186.06 | [$139.54 - $232.57] | 42 |
| Cost for transfer of critically ill patients to the south | Triangular | $1,535.02 | [$1,151.26 - $1,918.77] | 2 |
| Cost of follow-up clinic visit after tuberculin test^†^ | Triangular | $60.63 | [$45.47 - $75.79] | 52, ^[[55]](#endnote-55)^ |
| Nurse assessment wage for 20-min visit | Triangular | $27.73 | [$20.80 - $34.67] | ^[[56]](#endnote-56)^ |
| Cost of return flight to Nunavut after treatment in Ottawa^†^ | Triangular | $10,659.36 | [$7,994.52 - $13,324.20] | 2 |
| Cost of a spontaneous sputum production | Triangular | $3.75 | [$2.81 - $4.69] | 44 |
| Cost of an analysis of a positive sputum | Triangular | $86.04 | [$64.53 - $107.55] | ^[[57]](#endnote-57)^ |
| Cost of an induced sputum production^†^ | Triangular | $103.13 | [$77.35 - $128.92] | 57 |
| Cost of tuberculin skin test^†^ | Triangular | $19.40 | [$14.55 - $24.25] | 52 |
| Immunity from previous disease | Beta | 0.55 | [0.50 - 0.84] | Dynamic model |
| Probability of having adverse event from TB treatment* | Beta | 0.05 | [0.01 - 0.1] | 52 |
| Probability of having adverse event from latent TB treatment* | Beta | 0.003 | [0.001 - 0.005] | 52,^[[58]](#endnote-58)^ |
| Probability of completing treatment for active TB* | Beta | 0.95 | [0.94 - 0.96] | 44 |
| Probability of completing treatment for latent TB* | Beta | 0.756 | [0.531 - 0.885] | 26 |
| Probability of diagnosis of TB disease among individual with active disease* | Beta | 0.9 | [0.8 - 1] | Assumption |
| Probability of latent TB infection diagnosis* | Beta | 0.25 | [0.2 - 0.9] | Assumption |
| Probability of death during TB treatment for those with no TB risk factors* | Beta | 0.017 | [0.013 - 0.021] | Dynamic model |
| Probability of an individual evaluated for TB in a remote community being sent to Iqaluit for further TB related work-up* | Beta | 0.04 | [0.04 - 0.05] | 44 |
| Probability of coming back for TST reading* | Beta | 0.86 | [0.80 - 0.90] | ^[[59]](#endnote-59)^ |
| Annual risk of TB infection among those with no TB risk factors^†^ | Beta | 0.0017 | [0.0013 - 0.0021] | Dynamic model |
| Probability of producing suitable sputum samples given CXR abnormality* | Beta | 0.82 | [0.747 - 0.896] | 44 |
| Probability of progression of recently acquired latent TB infection to TB disease for those with no TB risk factors^†^* | Beta | 0.0018/yr for 2 yrs | [0.00135 - 0.00225] | Dynamic model |
| Probability of reactivation of old-standing latent TB infection to TB disease for those with no TB risk factors^†^* | Beta | 0.0005/yr | [0.0004 – 0.0007] | ^[[60]](#endnote-60)^.^[[61]](#endnote-61)^,^[[62]](#endnote-62)^ |
| Probability of cure among those who complete treatment for active TB* | Beta | 0.95 | [0.9 - 1] | Assumption |
| Probability of cure among those who complete treatment for latent TB* | Beta | 0.9 | [0.875 - 0.925] | ^[[63]](#endnote-63)^ |
| Probability of spontaneous recovery from active TB without treatment* | Beta | 0.25 | [0.2 - 0.3] | 6 |
| Probability of living in a remote community without a hospital* | Beta | 0.21 | [0.16 - 0.26] | ^[[64]](#endnote-64)^ |
| Relative risk of acquiring TB infection among smokers compared to non-smokers | Lognormal | 1.91 | [1.60 - 2.27] | ^[[65]](#endnote-65)^ |
| Relative risk of progression to active TB among smokers compared to non-smokers | Lognormal | 1.50 | [1.26 - 1.74] | 65 |
| Relative risk of death among smokers compared to non-smokers | Lognormal | 2.6 | [1.8 - 3.6] | ^[[66]](#endnote-66)^ |
| Relative risk of acquiring TB infection among heavy drinkers compared to non-heavy drinkers | Lognormal | 2.94 | [1.89 - 4.59] | 66 |
| Relative risk of progression to active TB among heavy drinkers compared to non-heavy drinkers | Lognormal | 4.21 | [2.73 - 6.48] | 66 |
| Relative risk of death among heavy drinkers compared to non-heavy drinkers | Lognormal | 2.4 | [1.1 - 5.3] | ^[[67]](#endnote-67)^ |
| Odds of acquiring TB infection among food insecure compared to food secure | Logistic | 2.1 | [1.0 - 4.3] | ^[[68]](#endnote-68)^ |
| Odds of progression to active TB among food insecure compared to food secure | Logistic | 2.38 | [1.6 - 3.1] | ^[[69]](#endnote-69)^ |
| Relative risk of acquiring TB infection among individuals in overcrowded housing compared to those in non-crowded housing | Lognormal | 1.5 | [1.1 - 2.0] | ^[[70]](#endnote-70)^ |
| Odds of progression to active TB among individuals in overcrowded housing compared to those in non-crowded housing | Logistic | 3.71 | [2.48 - 7.59] | ^[[71]](#endnote-71)^ |
| Annual percent decrease in number of individuals living in crowded dwellings  due to reduction strategy | Beta | 0.46 | [0.33 - 0.59] | 38 |
| Annual percent decrease in number of heavy drinkers due to reduction strategy | Beta | 0.28 | [0.23 - 0.34] | 40 |
| Relative risk of smoking cessation with reduction strategy vs. without | Lognormal | 2.36 | [1.01 - 5.53] | 30 |
| Odds of being food secure with reduction strategy vs. without | Logistic | 2.42 | [1.35 – 5.5] ^#^ | 41 |
| Number of days of hospitalization in case of suspicion of active disease before diagnosis results | Uniform | 3 | [2 - 4] | 44 |
| Hospitalization days at initiation | Uniform | 14 | [10 - 18] | 24 |
| Utility score for individual with active disease treated | Triangular | 0.85 | [0.8 - 0.9] | ^[[72]](#endnote-72)^ |
| Utility score for individual with active disease untreated | Triangular | 0.68 | [0.65 - 0.7] | ^[[73]](#endnote-73)^ |
| Utility score for individual with latent disease treated | Triangular | 0.97 | [0.95 - 1] | 72 |
| Utility score for individual with latent disease untreated | Triangular | 1 | [0.99 - 1] | Assumption |

^†^ Range: ±25% from the point estimate

* All probabilities are one-time, unless otherwise specified

^#^ 95% confidence interval from literature is [2.42 - 176.08]. We used the lower bound from literature as our point estimate

**Results**

The following figures illustrate the results of univariate sensitivity analysis, where the most influential drivers of TB incidence and TB-related health system and intervention costs were identified.

In Supplemental Figures 4-9, we simultaneously considered the status quo and all four TB risk factor reduction strategies, thereby identifying the most influential variables, on average, over all five scenarios.

**Supplemental Figure 4:**

**Univariate sensitivity analysis (cost per person for the combined food insecurity and tobacco reduction strategy)**

**
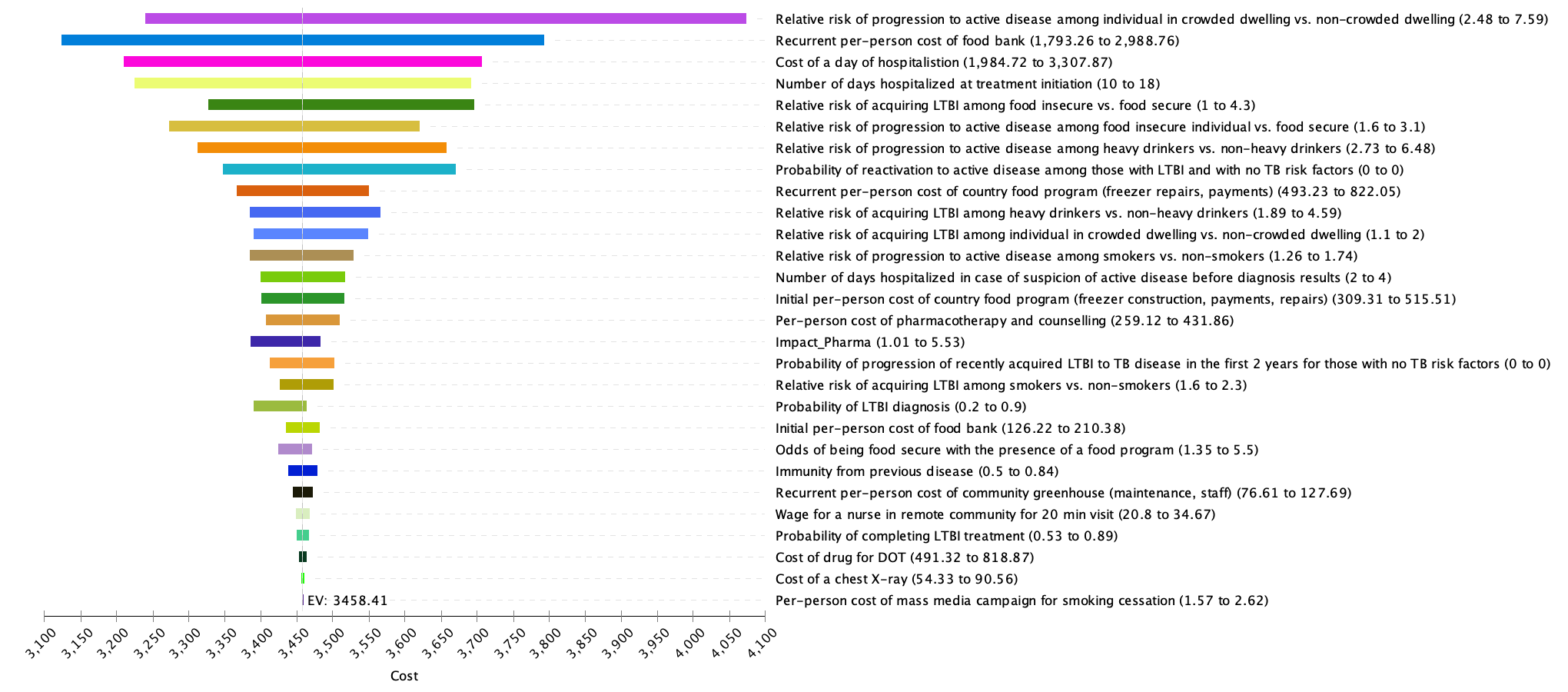
**

**Supplemental Figure 5:**

**Univariate sensitivity analysis (TB incidence per person for the combined food insecurity and tobacco reduction strategy)**

**
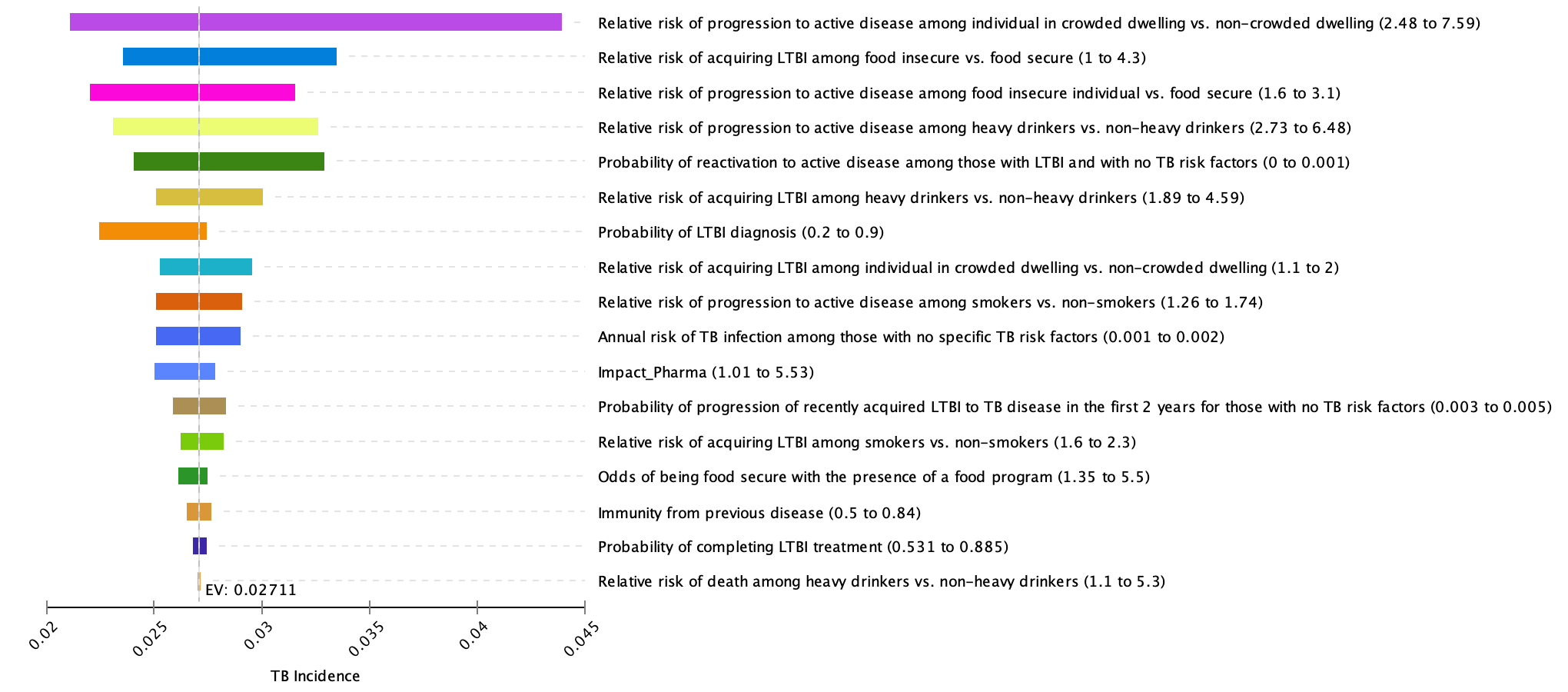
**

**Supplemental Figure 6:**

**Univariate sensitivity analysis (incremental cost per TB case averted: smoking strategy vs. status quo)**

**
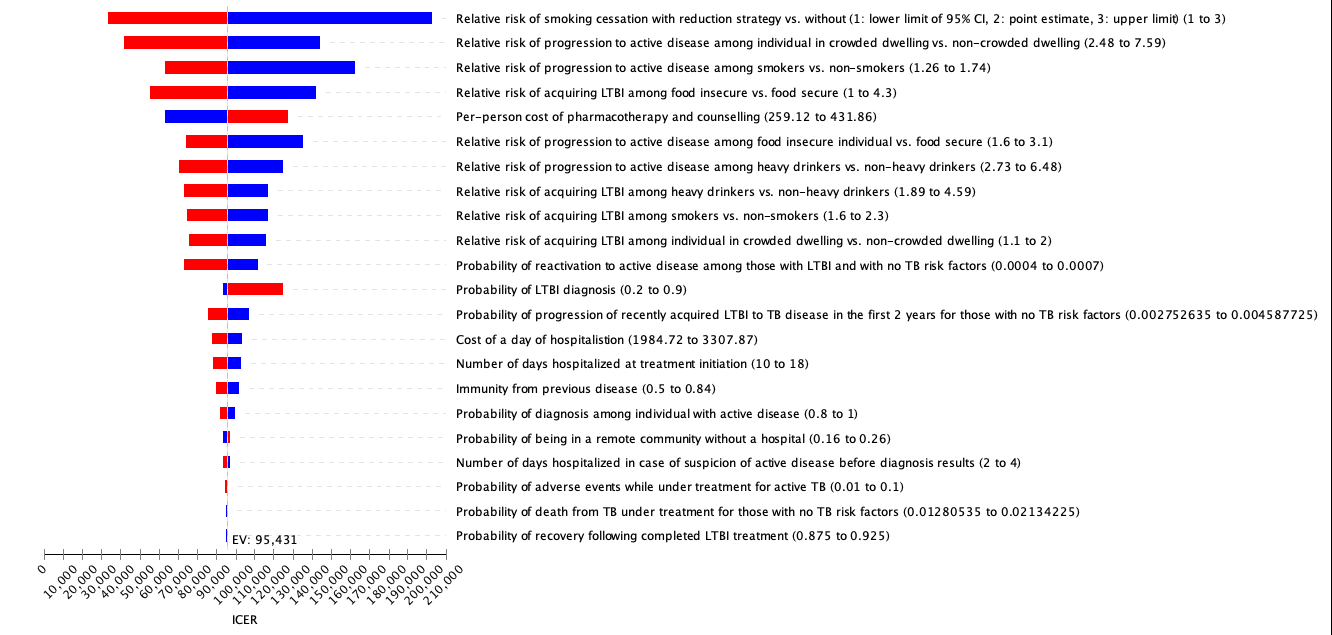
**

*Blue represents low parameter values and red represents high parameter values*

ICER = (Cost in intervention scenario – Cost in status-quo scenario) $\div$ (TB incidence in status-quo scenario – TB incidence in intervention scenario)

**Supplemental Figure 7:**

**Univariate sensitivity analysis (incremental cost per TB case averted: heavy drinking strategy vs. status quo)**

**
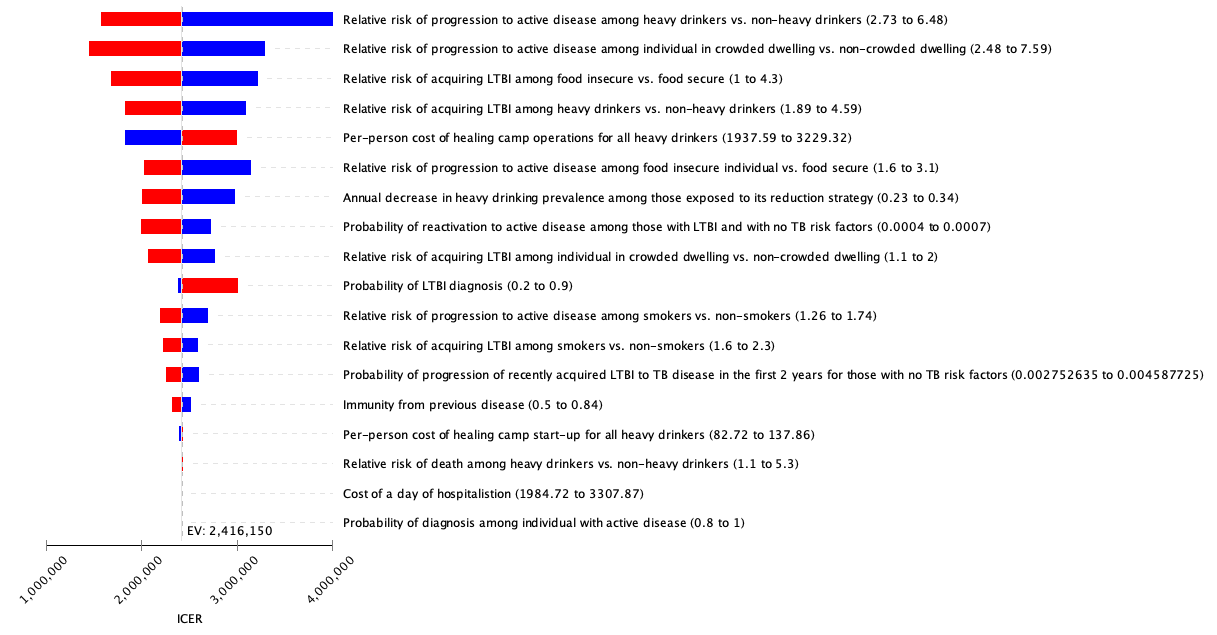
**

**Supplemental Figure 8:**

**Univariate sensitivity analysis (incremental cost per TB case averted: food insecurity strategy vs. status quo)**

**
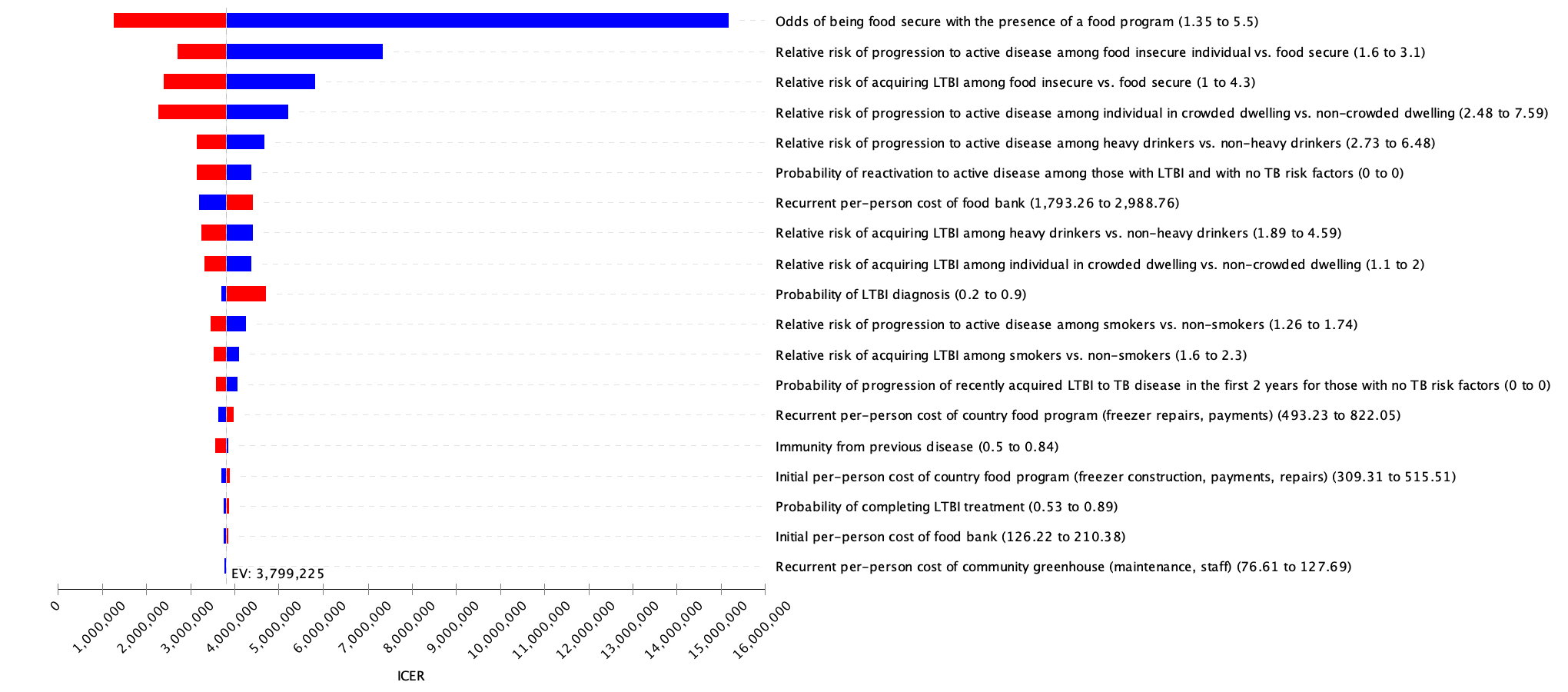
**

**Supplemental Figure 9:**

**Univariate sensitivity analysis (incremental cost per TB case averted: overcrowding strategy vs. status quo)**


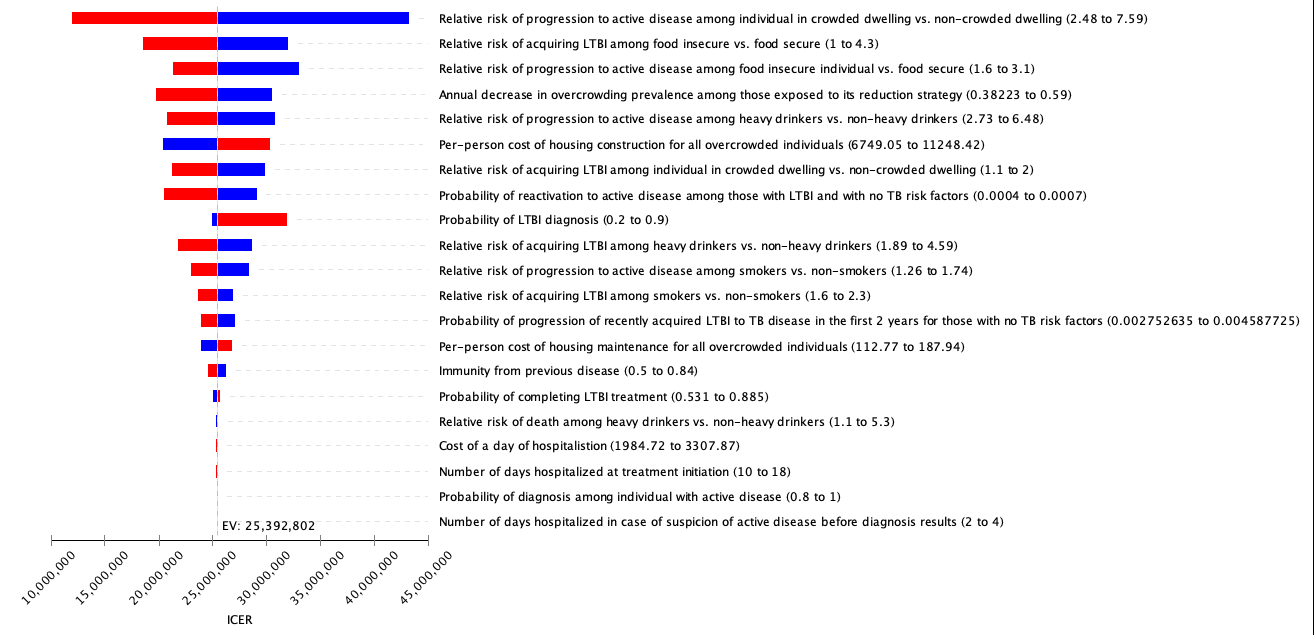


**Supplemental Figure 10:**

**Probabilistic sensitivity analysis, incremental cost per Quality Adjusted Life Year (QALY) gained compared to status quo**

**
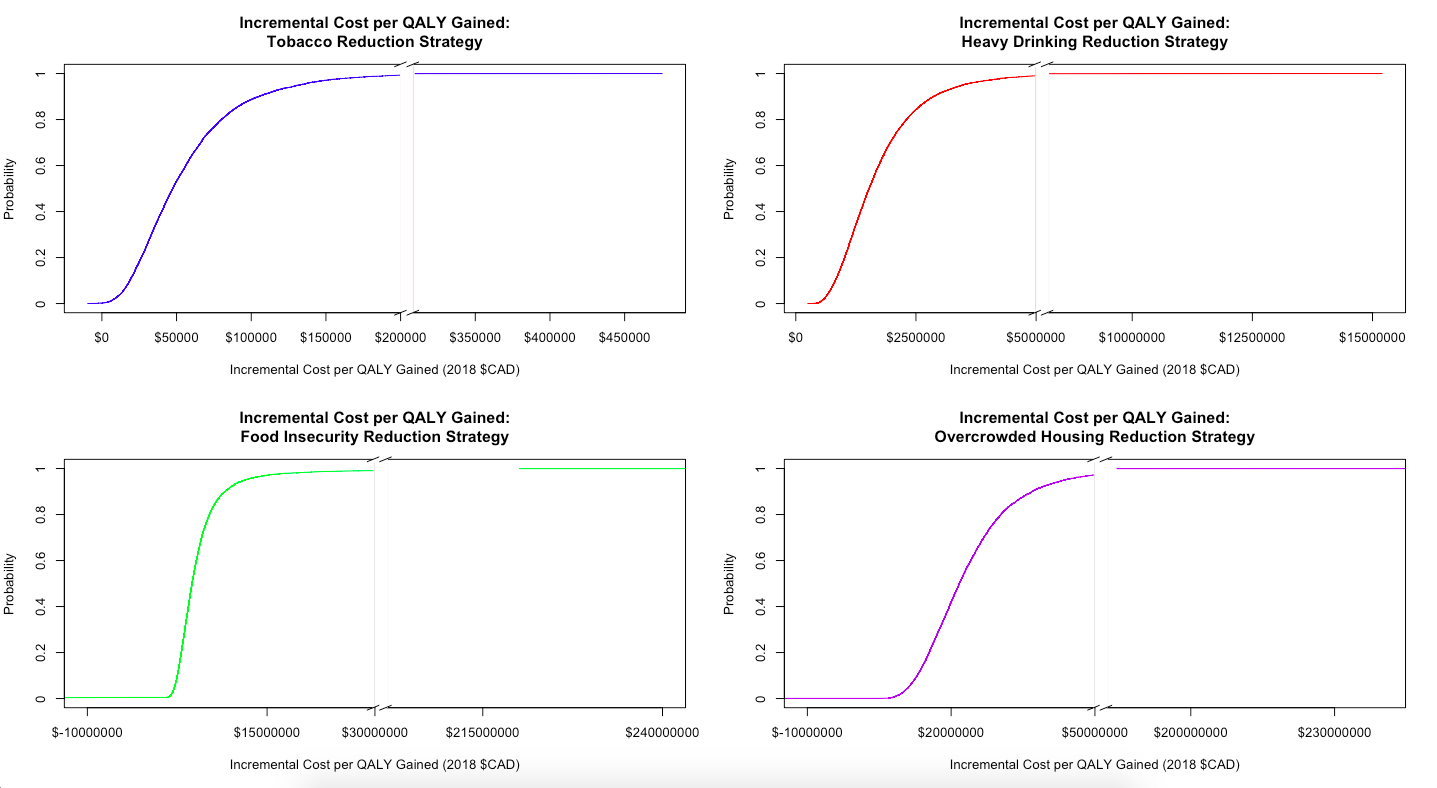
**

# SLIR Model Equations

## Strata and Corresponding Risk Factors:

- Stratum 1: Smoking, heavy drinking, food insecurity, overcrowding
- Stratum 2: Smoking, heavy drinking, food insecurity
- Stratum 3: Smoking, heavy drinking, overcrowding
- Stratum 4: Smoking, heavy drinking
- Stratum 5: Smoking, food insecurity, overcrowding
- Stratum 6: Smoking, food insecurity
- Stratum 7: Smoking, overcrowding
- Stratum 8: Smoking
- Stratum 9: Heavy drinking, food insecurity, overcrowding
- Stratum 10: Heavy drinking, food insecurity
- Stratum 11: Heavy drinking, overcrowding
- Stratum 12: Heavy drinking
- Stratum 13: Food insecurity, overcrowding
- Stratum 14: Food insecurity
- Stratum 15: Overcrowding
- Stratum 16: No risk factors

## TB Health States:

For *x* in {1, 2, 3, …, 16},

- $S_{x}=Susceptible$
- ${LS}_{x}=Latent Slow$
- ${LF}_{x}=Latent Fast$
- $I_{x}=Infectious$
- ${RL}_{x}=Recovered from Latent Disease$
- ${RA}_{x}=Recovered from Active Disease$
- ${RS}_{x}=Spontaneously Recovered$
- ${RLF}_{x}=Reinfected from Latent$

## Initial Conditions:

| $\boldsymbol{S}_{\boldsymbol{1}}\boldsymbol{(0)=5941.11}$  $\boldsymbol{LS}_{\boldsymbol{1}}\boldsymbol{(0)=4894.23}$  $\boldsymbol{LF}_{\boldsymbol{1}}\left( \boldsymbol{0} \right)\boldsymbol{=6872.43}$  $\boldsymbol{I}_{\boldsymbol{1}}\left( \boldsymbol{0} \right)\boldsymbol{=}\mathbf{10716.2}$  $\boldsymbol{RL}_{\boldsymbol{1}}\left( \boldsymbol{0} \right)\boldsymbol{=}\mathbf{0}$  $\boldsymbol{RA}_{\boldsymbol{1}}\left( \boldsymbol{0} \right)\boldsymbol{=}\mathbf{0}$  $\boldsymbol{RS}_{\boldsymbol{1}}\left( \boldsymbol{0} \right)\boldsymbol{=2789.16}$  $\boldsymbol{RLF}_{\boldsymbol{1}}\left( \boldsymbol{0} \right)\boldsymbol{=}\mathbf{12533.9}$ | $\boldsymbol{S}_{\boldsymbol{2}}\boldsymbol{(0)=}\mathbf{2789.16}$  $\boldsymbol{LS}_{\boldsymbol{2}}\boldsymbol{(0)=5865.87}$  $\boldsymbol{LF}_{\boldsymbol{2}}\left( \boldsymbol{0} \right)\boldsymbol{=3761.44}$  $\boldsymbol{I}_{\boldsymbol{2}}\left( \boldsymbol{0} \right)\boldsymbol{=2190.98}$  $\boldsymbol{RL}_{\boldsymbol{2}}\left( \boldsymbol{0} \right)\boldsymbol{=}\mathbf{0}$  $\boldsymbol{RA}_{\boldsymbol{2}}\left( \boldsymbol{0} \right)\boldsymbol{=}\mathbf{0}$  $\boldsymbol{RS}_{\boldsymbol{2}}\left( \boldsymbol{0} \right)\boldsymbol{=822.39}$  $\boldsymbol{RLF}_{\boldsymbol{2}}\left( \boldsymbol{0} \right)\boldsymbol{=}\mathbf{11632.2}$ | $\boldsymbol{S}_{\boldsymbol{3}}\boldsymbol{(0)=}\mathbf{5289.18}$  $\boldsymbol{LS}_{\boldsymbol{3}}\boldsymbol{(0)=8024.79}$  $\boldsymbol{LF}_{\boldsymbol{3}}\left( \boldsymbol{0} \right)\boldsymbol{=4660.25}$  $\boldsymbol{I}_{\boldsymbol{3}}\left( \boldsymbol{0} \right)\boldsymbol{=3658.26}$  $\boldsymbol{RL}_{\boldsymbol{3}}\left( \boldsymbol{0} \right)\boldsymbol{=}\mathbf{0}$  $\boldsymbol{RA}_{\boldsymbol{3}}\left( \boldsymbol{0} \right)\boldsymbol{=}\mathbf{0}$  $\boldsymbol{RS}_{\boldsymbol{3}}\left( \boldsymbol{0} \right)\boldsymbol{=}\mathbf{1886.06}$  $\boldsymbol{RLF}_{\boldsymbol{3}}\left( \boldsymbol{0} \right)\boldsymbol{=}\mathbf{10854.5}$ | $\boldsymbol{S}_{\boldsymbol{4}}\boldsymbol{(0)=}\mathbf{2610.96}$  $\boldsymbol{LS}_{\boldsymbol{4}}\boldsymbol{(0)=7541.09}$  $\boldsymbol{LF}_{\boldsymbol{4}}\left( \boldsymbol{0} \right)\boldsymbol{=2276.99}$  $\boldsymbol{I}_{\boldsymbol{4}}\left( \boldsymbol{0} \right)\boldsymbol{=626.893}$  $\boldsymbol{RL}_{\boldsymbol{4}}\left( \boldsymbol{0} \right)\boldsymbol{=}\mathbf{0}$  $\boldsymbol{RA}_{\boldsymbol{4}}\left( \boldsymbol{0} \right)\boldsymbol{=}\mathbf{0}$  $\boldsymbol{RS}_{\boldsymbol{4}}\left( \boldsymbol{0} \right)\boldsymbol{=454.696}$  $\boldsymbol{RLF}_{\boldsymbol{4}}\left( \boldsymbol{0} \right)\boldsymbol{=}\mathbf{7556.37}$ |
| --- | --- | --- | --- |
| $\boldsymbol{S}_{\boldsymbol{5}}\boldsymbol{(0)=33771.1}$  $\boldsymbol{LS}_{\boldsymbol{5}}\boldsymbol{(0)=8}\mathbf{1456.6}$  $\boldsymbol{LF}_{\boldsymbol{5}}\left( \boldsymbol{0} \right)\boldsymbol{=34233.6}$  $\boldsymbol{I}_{\boldsymbol{5}}\left( \boldsymbol{0} \right)\boldsymbol{=}\mathbf{23401.6}$  $\boldsymbol{RL}_{\boldsymbol{5}}\left( \boldsymbol{0} \right)\boldsymbol{=}\mathbf{0}$  $\boldsymbol{RA}_{\boldsymbol{5}}\left( \boldsymbol{0} \right)\boldsymbol{=}\mathbf{0}$  $\boldsymbol{RS}_{\boldsymbol{5}}\left( \boldsymbol{0} \right)\boldsymbol{=16189.4}$  $\boldsymbol{RLF}_{\boldsymbol{5}}\left( \boldsymbol{0} \right)\boldsymbol{=}\mathbf{87712.1}$ | $\boldsymbol{S}_{\boldsymbol{6}}\boldsymbol{(0)=}\mathbf{21983.5}$  $\boldsymbol{LS}_{\boldsymbol{6}}\boldsymbol{(0)=68966.7}$  $\boldsymbol{LF}_{\boldsymbol{6}}\left( \boldsymbol{0} \right)\boldsymbol{=14797.4}$  $\boldsymbol{I}_{\boldsymbol{6}}\left( \boldsymbol{0} \right)\boldsymbol{=3888.62}$  $\boldsymbol{RL}_{\boldsymbol{6}}\left( \boldsymbol{0} \right)\boldsymbol{=}\mathbf{0}$  $\boldsymbol{RA}_{\boldsymbol{6}}\left( \boldsymbol{0} \right)\boldsymbol{=}\mathbf{0}$  $\boldsymbol{RS}_{\boldsymbol{6}}\left( \boldsymbol{0} \right)\boldsymbol{=3721.78}$  $\boldsymbol{RLF}_{\boldsymbol{6}}\left( \boldsymbol{0} \right)\boldsymbol{=}\mathbf{51349}$ | $\boldsymbol{S}_{\boldsymbol{7}}\boldsymbol{(0)=39683.9}$  $\boldsymbol{LS}_{\boldsymbol{7}}\boldsymbol{(0)=}\mathbf{91021.1}$  $\boldsymbol{LF}_{\boldsymbol{7}}\left( \boldsymbol{0} \right)\boldsymbol{=17784}$  $\boldsymbol{I}_{\boldsymbol{7}}\left( \boldsymbol{0} \right)\boldsymbol{=6591.61}$  $\boldsymbol{RL}_{\boldsymbol{7}}\left( \boldsymbol{0} \right)\boldsymbol{=}\mathbf{0}$  $\boldsymbol{RA}_{\boldsymbol{7}}\left( \boldsymbol{0} \right)\boldsymbol{=}\mathbf{0}$  $\boldsymbol{RS}_{\boldsymbol{7}}\left( \boldsymbol{0} \right)\boldsymbol{=8256.13}$  $\boldsymbol{RLF}_{\boldsymbol{7}}\left( \boldsymbol{0} \right)\boldsymbol{=4}\mathbf{7810.3}$ | $\boldsymbol{S}_{\boldsymbol{8}}\boldsymbol{(0)=}\mathbf{29410.7}$  $\boldsymbol{LS}_{\boldsymbol{8}}\boldsymbol{(0)=64319.1}$  $\boldsymbol{LF}_{\boldsymbol{8}}\left( \boldsymbol{0} \right)\boldsymbol{=10036.9}$  $\boldsymbol{I}_{\boldsymbol{8}}\left( \boldsymbol{0} \right)\boldsymbol{=1001.16}$  $\boldsymbol{RL}_{\boldsymbol{8}}\left( \boldsymbol{0} \right)\boldsymbol{=}\mathbf{0}$  $\boldsymbol{RA}_{\boldsymbol{8}}\left( \boldsymbol{0} \right)\boldsymbol{=}\mathbf{0}$  $\boldsymbol{RS}_{\boldsymbol{8}}\left( \boldsymbol{0} \right)\boldsymbol{=1647.73}$  $\boldsymbol{RLF}_{\boldsymbol{8}}\left( \boldsymbol{0} \right)\boldsymbol{=}\mathbf{22997.4}$ |
| $\boldsymbol{S}_{\boldsymbol{9}}\left( \boldsymbol{0} \right)\boldsymbol{=592.226}$  $\boldsymbol{LS}_{\boldsymbol{9}}\boldsymbol{(0)=854.923}$  $\boldsymbol{LF}_{\boldsymbol{9}}\left( \boldsymbol{0} \right)\boldsymbol{=459.591}$  $\boldsymbol{I}_{\boldsymbol{9}}\left( \boldsymbol{0} \right)\boldsymbol{=1059.14}$  $\boldsymbol{RL}_{\boldsymbol{9}}\left( \boldsymbol{0} \right)\boldsymbol{=}\mathbf{0}$  $\boldsymbol{RA}_{\boldsymbol{9}}\left( \boldsymbol{0} \right)\boldsymbol{=}\mathbf{0}$  $\boldsymbol{RS}_{\boldsymbol{9}}\left( \boldsymbol{0} \right)\boldsymbol{=499.199}$  $\boldsymbol{RLF}_{\boldsymbol{9}}\left( \boldsymbol{0} \right)\boldsymbol{=}\mathbf{1395.92}$ | $\boldsymbol{S}_{\boldsymbol{10}}\boldsymbol{(0)=}\mathbf{329.342}$  $\boldsymbol{LS}_{\boldsymbol{10}}\boldsymbol{(0)=}\mathbf{939.299}$  $\boldsymbol{LF}_{\boldsymbol{10}}\left( \boldsymbol{0} \right)\boldsymbol{=288.423}$  $\boldsymbol{I}_{\boldsymbol{10}}\left( \boldsymbol{0} \right)\boldsymbol{=}\mathbf{213.745}$  $\boldsymbol{RL}_{\boldsymbol{10}}\left( \boldsymbol{0} \right)\boldsymbol{=}\mathbf{0}$  $\boldsymbol{RA}_{\boldsymbol{10}}\left( \boldsymbol{0} \right)\boldsymbol{=}\mathbf{0}$  $\boldsymbol{RS}_{\boldsymbol{10}}\left( \boldsymbol{0} \right)\boldsymbol{=}\mathbf{142.334}$  $\boldsymbol{RLF}_{\boldsymbol{10}}\left( \boldsymbol{0} \right)\boldsymbol{=}\mathbf{1065.86}$ | $\boldsymbol{S}_{\boldsymbol{11}}\boldsymbol{(0)=}\mathbf{625.952}$  $\boldsymbol{LS}_{\boldsymbol{11}}\boldsymbol{(0)=}\mathbf{1215.85}$  $\boldsymbol{LF}_{\boldsymbol{11}}\left( \boldsymbol{0} \right)\boldsymbol{=}\mathbf{339.43}$  $\boldsymbol{I}_{\boldsymbol{11}}\left( \boldsymbol{0} \right)\boldsymbol{=}\mathbf{349.048}$  $\boldsymbol{RL}_{\boldsymbol{11}}\left( \boldsymbol{0} \right)\boldsymbol{=}\mathbf{0}$  $\boldsymbol{RA}_{\boldsymbol{11}}\left( \boldsymbol{0} \right)\boldsymbol{=}\mathbf{0}$  $\boldsymbol{RS}_{\boldsymbol{11}}\left( \boldsymbol{0} \right)\boldsymbol{=}\mathbf{311.541}$  $\boldsymbol{RLF}_{\boldsymbol{11}}\left( \boldsymbol{0} \right)\boldsymbol{=}\mathbf{977.177}$ | $\boldsymbol{S}_{\boldsymbol{12}}\boldsymbol{(0)=}\mathbf{406.659}$  $\boldsymbol{LS}_{\boldsymbol{12}}\boldsymbol{(0)=}\mathbf{1035.03}$  $\boldsymbol{LF}_{\boldsymbol{12}}\left( \boldsymbol{0} \right)\boldsymbol{=197.975}$  $\boldsymbol{I}_{\boldsymbol{12}}\left( \boldsymbol{0} \right)\boldsymbol{=}\mathbf{59.3647}$  $\boldsymbol{RL}_{\boldsymbol{12}}\left( \boldsymbol{0} \right)\boldsymbol{=}\mathbf{0}$  $\boldsymbol{RA}_{\boldsymbol{12}}\left( \boldsymbol{0} \right)\boldsymbol{=}\mathbf{0}$  $\boldsymbol{RS}_{\boldsymbol{12}}\left( \boldsymbol{0} \right)\boldsymbol{=}\mathbf{71.9426}$  $\boldsymbol{RLF}_{\boldsymbol{12}}\left( \boldsymbol{0} \right)\boldsymbol{=}\mathbf{570.026}$ |
| $\boldsymbol{S}_{\boldsymbol{13}}\boldsymbol{(0)=}\mathbf{4970.69}$  $\boldsymbol{LS}_{\boldsymbol{13}}\boldsymbol{(0)=}\mathbf{11589.1}$  $\boldsymbol{LF}_{\boldsymbol{13}}\left( \boldsymbol{0} \right)\boldsymbol{=2257.67}$  $\boldsymbol{I}_{\boldsymbol{13}}\left( \boldsymbol{0} \right)\boldsymbol{=}\mathbf{1886.14}$  $\boldsymbol{RL}_{\boldsymbol{13}}\left( \boldsymbol{0} \right)\boldsymbol{=}\mathbf{0}$  $\boldsymbol{RA}_{\boldsymbol{13}}\left( \boldsymbol{0} \right)\boldsymbol{=}\mathbf{0}$  $\boldsymbol{RS}_{\boldsymbol{13}}\left( \boldsymbol{0} \right)\boldsymbol{=}\mathbf{2192.39}$  $\boldsymbol{RLF}_{\boldsymbol{13}}\left( \boldsymbol{0} \right)\boldsymbol{=}\mathbf{6963}$ | $\boldsymbol{S}_{\boldsymbol{14}}\boldsymbol{(0)=}\mathbf{3813.07}$  $\boldsymbol{LS}_{\boldsymbol{14}}\boldsymbol{(0)=}\mathbf{8794.48}$  $\boldsymbol{LF}_{\boldsymbol{14}}\left( \boldsymbol{0} \right)\boldsymbol{=1399.75}$  $\boldsymbol{I}_{\boldsymbol{14}}\left( \boldsymbol{0} \right)\boldsymbol{=}\mathbf{306.201}$  $\boldsymbol{RL}_{\boldsymbol{14}}\left( \boldsymbol{0} \right)\boldsymbol{=}\mathbf{0}$  $\boldsymbol{RA}_{\boldsymbol{14}}\left( \boldsymbol{0} \right)\boldsymbol{=}\mathbf{0}$  $\boldsymbol{RS}_{\boldsymbol{14}}\left( \boldsymbol{0} \right)\boldsymbol{=}\mathbf{471.486}$  $\boldsymbol{RLF}_{\boldsymbol{14}}\left( \boldsymbol{0} \right)\boldsymbol{=}\mathbf{3516.01}$ | $\boldsymbol{S}_{\boldsymbol{15}}\boldsymbol{(0)=}\mathbf{6474.44}$  $\boldsymbol{LS}_{\boldsymbol{15}}\boldsymbol{(0)=}\mathbf{1609.9}$  $\boldsymbol{LF}_{\boldsymbol{15}}\left( \boldsymbol{0} \right)\boldsymbol{=10791.8}$  $\boldsymbol{I}_{\boldsymbol{15}}\left( \boldsymbol{0} \right)\boldsymbol{=}\mathbf{514.64}$  $\boldsymbol{RL}_{\boldsymbol{15}}\left( \boldsymbol{0} \right)\boldsymbol{=}\mathbf{0}$  $\boldsymbol{RA}_{\boldsymbol{15}}\left( \boldsymbol{0} \right)\boldsymbol{=}\mathbf{0}$  $\boldsymbol{RS}_{\boldsymbol{15}}\left( \boldsymbol{0} \right)\boldsymbol{=}\mathbf{990.196}$  $\boldsymbol{RLF}_{\boldsymbol{15}}\left( \boldsymbol{0} \right)\boldsymbol{=}\mathbf{3079.98}$ | $\boldsymbol{S}_{\boldsymbol{16}}\boldsymbol{(0)=}\mathbf{5021.4}$  $\boldsymbol{LS}_{\boldsymbol{16}}\boldsymbol{(0)=}\mathbf{6874.73}$  $\boldsymbol{LF}_{\boldsymbol{16}}\left( \boldsymbol{0} \right)\boldsymbol{=916.229}$  $\boldsymbol{I}_{\boldsymbol{16}}\left( \boldsymbol{0} \right)\boldsymbol{=}\mathbf{76.8556}$  $\boldsymbol{RL}_{\boldsymbol{16}}\left( \boldsymbol{0} \right)\boldsymbol{=}\mathbf{0}$  $\boldsymbol{RA}_{\boldsymbol{16}}\left( \boldsymbol{0} \right)\boldsymbol{=}\mathbf{0}$  $\boldsymbol{RS}_{\boldsymbol{16}}\left( \boldsymbol{0} \right)\boldsymbol{=}\mathbf{183.286}$  $\boldsymbol{RLF}_{\boldsymbol{16}}\left( \boldsymbol{0} \right)\boldsymbol{=}\mathbf{1306.51}$ |

## Difference Equations:

For *x* in {1, 2, 3, …, 16},

1. $S_{x}\left( t+dt \right)=S_{x}\left( t \right)- S_{x}\left( t \right)*\left( b\frac{I_{1}\left( t \right)}{N}+b\frac{I_{2}\left( t \right)}{N}+b\frac{I_{3}\left( t \right)}{N}+b\frac{I_{4}\left( t \right)}{N}+b\frac{I_{5}\left( t \right)}{N}+b\frac{I_{6}\left( t \right)}{N}+b\frac{I_{7}\left( t \right)}{N}+b\frac{I_{8}\left( t \right)}{N}+b\frac{I_{9}\left( t \right)}{N}+b\frac{I_{10}\left( t \right)}{N}+b\frac{I_{11}\left( t \right)}{N}+b\frac{I_{12}\left( t \right)}{N}+b\frac{I_{13}\left( t \right)}{N}+b\frac{I_{14}\left( t \right)}{N}+b\frac{I_{15}\left( t \right)}{N}+b\frac{I_{16}\left( t \right)}{N} \right)*{EE}_{i}-S_{x}(t)*d_{rate}+{(S}_{x}(t)+LS_{x}(t)+I_{x}(t)+RL_{x}(t)+LF_{x}(t)+RA_{x}(t)+RS_{x}(t)+RLF_{x}(t))*d_{rate}+ I_{x}(t)*z_{x}*\left( 1-cd \right)+I_{x}(t)*cd*zd_{x}-S_{x}(t)*transition$
2. $LS_{x}\left( t+dt \right)= LS_{x}\left( t \right)-LS_{x}\left( t \right)*prot_{ltbi}*cd_{latent}-LS_{x}\left( t \right)*v_{x}-LS_{x}\left( t \right)*d_{rate}+LF_{x}\left( t \right)*t-{LS}_{x}\left( t \right)*\left( b\frac{I_{1}\left( t \right)}{N}+b\frac{I_{2}\left( t \right)}{N}+b\frac{I_{3}\left( t \right)}{N}+b\frac{I_{4}\left( t \right)}{N}+b\frac{I_{5}\left( t \right)}{N}+b\frac{I_{6}\left( t \right)}{N}+b\frac{I_{7}\left( t \right)}{N}+b\frac{I_{8}\left( t \right)}{N}+b\frac{I_{9}\left( t \right)}{N}+b\frac{I_{10}\left( t \right)}{N}+b\frac{I_{11}\left( t \right)}{N}+b\frac{I_{12}\left( t \right)}{N}+b\frac{I_{13}\left( t \right)}{N}+b\frac{I_{14}\left( t \right)}{N}+b\frac{I_{15}\left( t \right)}{N}+b\frac{I_{16}\left( t \right)}{N} \right)*{EE}_{i}+RLF_{x}\left( t \right)*t-LS_{x}(t)*transition$
3. $LF_{x}\left( t+dt \right)= LF_{x}\left( t \right)-LF_{x}\left( t \right)*t+S_{x}\left( t \right)*\left( b\frac{I_{1}\left( t \right)}{N}+b\frac{I_{2}\left( t \right)}{N}+b\frac{I_{3}\left( t \right)}{N}+b\frac{I_{4}\left( t \right)}{N}+b\frac{I_{5}\left( t \right)}{N}+b\frac{I_{6}\left( t \right)}{N}+b\frac{I_{7}\left( t \right)}{N}+b\frac{I_{8}\left( t \right)}{N}+b\frac{I_{9}\left( t \right)}{N}+b\frac{I_{10}\left( t \right)}{N}+b\frac{I_{11}\left( t \right)}{N}+b\frac{I_{12}\left( t \right)}{N}+b\frac{I_{13}\left( t \right)}{N}+b\frac{I_{14}\left( t \right)}{N}+b\frac{I_{15}\left( t \right)}{N}+b\frac{I_{16}\left( t \right)}{N} \right)*{EE}_{i}-LF_{x}\left( t \right)*p_{x}-LF_{x}\left( t \right)*prot_{ltbi}*cd_{latent}-LF_{x}\left( t \right)*d_{rate}-LF_{x}(t)*transition$
4. $I_{x}\left( t+dt \right)=I_{x}\left( t \right)-I_{x}\left( t \right)*z_{x}*\left( 1-cd \right)+{RS}_{x}\left( t \right)*{vSR}_{x}-I_{x}\left( t \right)*d+{RA}_{x}\left( t \right)*{vAT}_{x}-I_{x}\left( t \right)*d_{rate}+{LF}_{x}\left( t \right)*p_{x}+ {LS}_{x}\left( t \right)*v_{x}-I_{x}\left( t \right)*{tx}_{rate}*cd*txs+{RLF}_{x}\left( t \right)*p_{x}*im-I_{x}\left( t \right)*cd*{zd}_{x}-I_{x}(t)*transition$
5. ${RL}_{x}\left( t+dt \right)={RL}_{x}\left( t \right)+LS_{x}\left( t \right)*prot_{ltbi}*cd_{latent}+LF_{x}\left( t \right)*prot_{ltbi}*cd_{latent}-RL_{x}\left( t \right)*d_{rate}-{RL}_{x}\left( t \right)*\left( b\frac{I_{1}\left( t \right)}{N}+b\frac{I_{2}\left( t \right)}{N}+b\frac{I_{3}\left( t \right)}{N}+b\frac{I_{4}\left( t \right)}{N}+b\frac{I_{5}\left( t \right)}{N}+b\frac{I_{6}\left( t \right)}{N}+b\frac{I_{7}\left( t \right)}{N}+b\frac{I_{8}\left( t \right)}{N}+b\frac{I_{9}\left( t \right)}{N}+b\frac{I_{10}\left( t \right)}{N}+b\frac{I_{11}\left( t \right)}{N}+b\frac{I_{12}\left( t \right)}{N}+b\frac{I_{13}\left( t \right)}{N}+b\frac{I_{14}\left( t \right)}{N}+b\frac{I_{15}\left( t \right)}{N}+b\frac{I_{16}\left( t \right)}{N} \right)*{EE}_{i}+RLF_{x}\left( t \right)*prot_{ltbi}*cd_{latent}-RL_{x}\left( t \right)*transition$
6. ${RA}_{x}\left( t+dt \right)={RA}_{x}\left( t \right)-RA_{x}\left( t \right)*d_{rate}-RA_{x}\left( t \right)*vAT_{x}+I_{x}\left( t \right)*tx_{rate}*cd*txs-{RA}_{x}\left( t \right)*\left( b\frac{I_{1}\left( t \right)}{N}+b\frac{I_{2}\left( t \right)}{N}+b\frac{I_{3}\left( t \right)}{N}+b\frac{I_{4}\left( t \right)}{N}+b\frac{I_{5}\left( t \right)}{N}+b\frac{I_{6}\left( t \right)}{N}+b\frac{I_{7}\left( t \right)}{N}+b\frac{I_{8}\left( t \right)}{N}+b\frac{I_{9}\left( t \right)}{N}+b\frac{I_{10}\left( t \right)}{N}+b\frac{I_{11}\left( t \right)}{N}+b\frac{I_{12}\left( t \right)}{N}+b\frac{I_{13}\left( t \right)}{N}+b\frac{I_{14}\left( t \right)}{N}+b\frac{I_{15}\left( t \right)}{N}+b\frac{I_{16}\left( t \right)}{N} \right)*{EE}_{i}-RA_{x}\left( t \right)*transition$
7. ${RS}_{x}\left( t+dt \right)={RS}_{x}\left( t \right)+I_{x}\left( t \right)*d-RS_{x}\left( t \right)*d_{rate}-RS_{x}\left( t \right)*vSR_{x}-{RS}_{x}\left( t \right)*\left( b\frac{I_{1}\left( t \right)}{N}+b\frac{I_{2}\left( t \right)}{N}+b\frac{I_{3}\left( t \right)}{N}+b\frac{I_{4}\left( t \right)}{N}+b\frac{I_{5}\left( t \right)}{N}+b\frac{I_{6}\left( t \right)}{N}+b\frac{I_{7}\left( t \right)}{N}+b\frac{I_{8}\left( t \right)}{N}+b\frac{I_{9}\left( t \right)}{N}+b\frac{I_{10}\left( t \right)}{N}+b\frac{I_{11}\left( t \right)}{N}+b\frac{I_{12}\left( t \right)}{N}+b\frac{I_{13}\left( t \right)}{N}+b\frac{I_{14}\left( t \right)}{N}+b\frac{I_{15}\left( t \right)}{N}+b\frac{I_{16}\left( t \right)}{N} \right)*{EE}_{i}-RS_{x}\left( t \right)*transition$
8. ${RLF}_{x}\left( t+dt \right)={RLF}_{x}\left( t \right)+{RA}_{x}\left( t \right)*\left( b\frac{I_{1}\left( t \right)}{N}+b\frac{I_{2}\left( t \right)}{N}+b\frac{I_{3}\left( t \right)}{N}+b\frac{I_{4}\left( t \right)}{N}+b\frac{I_{5}\left( t \right)}{N}+b\frac{I_{6}\left( t \right)}{N}+b\frac{I_{7}\left( t \right)}{N}+b\frac{I_{8}\left( t \right)}{N}+b\frac{I_{9}\left( t \right)}{N}+b\frac{I_{10}\left( t \right)}{N}+b\frac{I_{11}\left( t \right)}{N}+b\frac{I_{12}\left( t \right)}{N}+b\frac{I_{13}\left( t \right)}{N}+b\frac{I_{14}\left( t \right)}{N}+b\frac{I_{15}\left( t \right)}{N}+b\frac{I_{16}\left( t \right)}{N} \right)*{EE}_{i}-RLF_{x}*p_{S}*im+{RS}_{x}\left( t \right)*\left( b\frac{I_{1}\left( t \right)}{N}+b\frac{I_{2}\left( t \right)}{N}+b\frac{I_{3}\left( t \right)}{N}+b\frac{I_{4}\left( t \right)}{N}+b\frac{I_{5}\left( t \right)}{N}+b\frac{I_{6}\left( t \right)}{N}+b\frac{I_{7}\left( t \right)}{N}+b\frac{I_{8}\left( t \right)}{N}+b\frac{I_{9}\left( t \right)}{N}+b\frac{I_{10}\left( t \right)}{N}+b\frac{I_{11}\left( t \right)}{N}+b\frac{I_{12}\left( t \right)}{N}+b\frac{I_{13}\left( t \right)}{N}+b\frac{I_{14}\left( t \right)}{N}+b\frac{I_{15}\left( t \right)}{N}+b\frac{I_{16}\left( t \right)}{N} \right)*{EE}_{i}+{RL}_{x}\left( t \right)*\left( b\frac{I_{1}\left( t \right)}{N}+b\frac{I_{2}\left( t \right)}{N}+b\frac{I_{3}\left( t \right)}{N}+b\frac{I_{4}\left( t \right)}{N}+b\frac{I_{5}\left( t \right)}{N}+b\frac{I_{6}\left( t \right)}{N}+b\frac{I_{7}\left( t \right)}{N}+b\frac{I_{8}\left( t \right)}{N}+b\frac{I_{9}\left( t \right)}{N}+b\frac{I_{10}\left( t \right)}{N}+b\frac{I_{11}\left( t \right)}{N}+b\frac{I_{12}\left( t \right)}{N}+b\frac{I_{13}\left( t \right)}{N}+b\frac{I_{14}\left( t \right)}{N}+b\frac{I_{15}\left( t \right)}{N}+b\frac{I_{16}\left( t \right)}{N} \right)*{EE}_{i}+{LS}_{x}\left( t \right)*\left( b\frac{I_{1}\left( t \right)}{N}+b\frac{I_{2}\left( t \right)}{N}+b\frac{I_{3}\left( t \right)}{N}+b\frac{I_{4}\left( t \right)}{N}+b\frac{I_{5}\left( t \right)}{N}+b\frac{I_{6}\left( t \right)}{N}+b\frac{I_{7}\left( t \right)}{N}+b\frac{I_{8}\left( t \right)}{N}+b\frac{I_{9}\left( t \right)}{N}+b\frac{I_{10}\left( t \right)}{N}+b\frac{I_{11}\left( t \right)}{N}+b\frac{I_{12}\left( t \right)}{N}+b\frac{I_{13}\left( t \right)}{N}+b\frac{I_{14}\left( t \right)}{N}+b\frac{I_{15}\left( t \right)}{N}+b\frac{I_{16}\left( t \right)}{N} \right)*{EE}_{i}-RLF_{x}\left( t \right)*prot_{ltbi}*cd_{latent}-RLF_{x}\left( t \right)*d_{rate}-RLF_{x}\left( t \right)*t-RLF_{x}\left( t \right)*transition$

## Infection Multipliers

$$EE_{i}=\left\{ \begin{aligned} 1.9 \times2.94 \times2.1 \times1.48 if in Stratum 1 \\ 1.9 \times2.94 \times2.1 if in Stratum 2 \\ 1.9 \times2.94 \times1.48 if in Stratum 3 \\ 1.9 \times2.94 if in Stratum 4 \\ 1.9 \times2.1\times1.48 if in Stratum 5 \\ 1.9 \times2.1 if in Stratum 6 \\ 1.9 \times1.48 if in Stratum 7 \\ 1.9 if in Stratum 8 \\ 2.94 \times2.1 \times1.48 if in Stratum 9 \\ 2.94 \times2.1 if in Stratum 10 \\ 2.94 \times1.48 if in Stratum 11 \\ 2.94 if in Stratum 12 \\ 2.1 \times1.48 if in Stratum 13 \\ 2.1 if in Stratum 14 \\ 1.48 if in Stratum 15 \\ 1 if in Stratum 16 \end{aligned} \right.$$

## Mortality Multipliers

$$E{Edeath}_{i}=\left\{ \begin{aligned} 2.6 \times2.4 if in Stratum 1 \\ 2.6 \times2.4 if in Stratum 2 \\ 2.6 \times2.4 if in Stratum 3 \\ 2.6 \times2.4 if in Stratum 4 \\ 2.6 if in Stratum 5 \\ 2.6 if in Stratum 6 \\ 2.6 if in Stratum 7 \\ 2.6 if in Stratum 8 \\ 2.4 if in Stratum 9 \\ 2.4 if in Stratum 10 \\ 2.4 if in Stratum 11 \\ 2.4 if in Stratum 12 \\ 1 if in Stratum 13 \\ 1 if in Stratum 14 \\ 1 if in Stratum 15 \\ 1 if in Stratum 16 \end{aligned} \right.$$

## Progression Multipliers

$$E{Eprogression}_{i}=\left\{ \begin{aligned} 1.5 \times4.21 \times2.38 \times3.71 if in Stratum 1 \\ 1.5 \times4.21 \times2.38 if in Stratum 2 \\ 1.5 \times4.21 \times3.71 if in Stratum 3 \\ 1.5 \times4.21 if in Stratum 4 \\ 1.5 \times2.38\times3.71 if in Stratum 5 \\ 1.5 \times2.38 if in Stratum 6 \\ 1.5 \times3.71 if in Stratum 7 \\ 1.5 if in Stratum 8 \\ 4.21 \times2.38 \times3.71 if in Stratum 9 \\ 4.21 \times2.38 if in Stratum 10 \\ 4.21 \times3.71 if in Stratum 11 \\ 4.21 if in Stratum 12 \\ 2.38 \times3.71 if in Stratum 13 \\ 2.38 if in Stratum 14 \\ 3.71 if in Stratum 15 \\ 1 if in Stratum 16 \end{aligned} \right.$$

## Parameter Values

### Pathogenetic Parameters (Stratum 16):

$p_{16}=if t<19 then 0.0091e^{-0.0188t} else if 19\leq t<52 then 0.00636666e^{-0.159(t-19)} else 0.000035095+0.0001(t-52)$

$$v_{16}=if t \leq52 then 0.001-0.0000096t else 0.0005$$

$$z_{16}=0.19\div\sum_{i=1}^{16} proportion of population in stratum i \times{EEdeath}_{i}$$

$${zd}_{16}=0.042 \div\sum_{i=1}^{16} proportion of population in stratum i \times{EEdeath}_{i}$$

### Pathogenetic Parameters (all other strata)

For *i* in {1, 2, 3, …, 16},

$$p_{i}=p_{16}*{EEprogression}_{i}$$

$$v_{i}=v_{16}*{EEprogression}_{i}$$

$$z_{i}=z_{16}*{EEdeath}_{i}$$

$${zd}_{i}=zd_{16}*{EEdeath}_{i}$$

### Other Parameters (Common to all strata)

$$t=0.25$$

$$d=0.25$$

$$im=0.55$$

$$cd_{latent}=if t<20 then 0 else if 20\leq t<45 then 0.0012\left( t-20 \right) else 0.03$$

$$cd= if t<20 then 0 else if 20\leq t<30 then 0.08\left( t-20 \right)+0.1 else 0.9$$

$$tx_{rate}=1$$

$$txs=0.97$$

$$prot_{ltbi}=0.4833$$

$$N=1000000 \left\{ total population \right\}$$

$$d_{rate}=0.000005t^{2}-0.0004t+0.023$$

$b=if t<19 then 0.925e^{-0.051t} else if 19\leq t<52 then 0.351003e^{-0.005\left( t-19 \right)} else 0.27613+0.032(t-52)$

$vSR=if t<19 then 0.025 else if 19\leq t<52 then 0.025e^{-0.05\left( t-19 \right)} else 0.00480125+0.0006(t-52)$

$vAT=if t<19 then 0.015 else if 19\leq t<52 then 0.015e^{-0.05\left( t-19 \right)} else 0.00288075+0.0006(t-52)$

## Transition Rates

*Dictate how individuals move between strata as prevalence of risk factors in the region changes over time*

$transition_{smoke}=if t<20 then 0 else if 20\leq t<68 then-0.000024t^{2}+0.002106t-0.033237 else 0.000129$

$transition_{drink}=0.0007$

$transition_{food}=if t<52 then 0 else if 52\leq t<66 then 0.001649841608t^{2}-0.200987885t+6.085236123 else -0.003599$

$transition_{rowd}=if t<49 then 0 else 0.19854-0.003364t$

# References

1. . Lönnroth K, Williams, BG, Stadlin S, Jaramillo E, Dye C. Alcohol use as a risk factor for tuberculosis – a systematic review. BMC Public Health. 2008;8:289. <https://bmcpublichealth.biomedcentral.com/track/pdf/10.1186/1471-2458-8-289> [↑](#endnote-ref-1)
2. . N’Diaye DS, Nsengiyumva NP, Uppal A, Oxlade O, Alvarez GG, Schwartzman K. The potential impact and cost-effectiveness of tobacco reduction strategies for tuberculosis prevention in Canadian Inuit communities. BMC Medicine. 2019;17(26). <https://bmcmedicine.biomedcentral.com/track/pdf/10.1186/s12916-019-1261-5> [↑](#endnote-ref-2)
3. . Rieder H. Epidemiologic basis of tuberculosis control. International Union Against Tuberculosis and Lung Disease. 1999 [↑](#endnote-ref-3)
4. . Canadian Human Mortality Database. Canada - Northwest Territories & Nunavut, Life expectancy at birth (period, 1x1). In: Demography Do, editor. Université de Montréal (Canada)2018. [www.demo.umontreal.ca/chmd/](http://www.demo.umontreal.ca/chmd/) [↑](#endnote-ref-4)
5. . LaFreniere M, Hussain H, He N, McGuire M. Tuberculosis in Canada: 2017. Can Commun Dis Rep 2019 Dec;45(2/3):68–74. <https://doi.org/10.14745/ccdr.v45i23a04> [↑](#endnote-ref-5)
6. .  Grzybowski S, Enarson DA. The fate of cases of pulmonary tuberculosis under various treatment programmes. Bull IUAT. 1978;53(2):70-5 [↑](#endnote-ref-6)
7. . Grzybowski S, Styblo K, Dorken E. Tuberculosis in eskimos. Tubercle. 1976;57(4):S1-S58 [↑](#endnote-ref-7)
8. . Grzybowski S, Dorken E. Tuberculosis in Inuit. Ecol Dis. 1983;2(2):145-8 [↑](#endnote-ref-8)
9. . Barsh RL. Canada’s Aboriginal peoples: Social integration or disintegration. The Canadian Journal of Native Studies. 1994;14(1):1-46 [↑](#endnote-ref-9)
10. . MacDonald N, Hébert PC, Stanbrook MB. Tuberculosis in Nunavut: a century of failure. CMAJ. 2011 [↑](#endnote-ref-10)
11. . Lee RS, Proulx J-F, Menzies D, Behr MA. Progression to tuberculosis disease increases with multiple exposures. Eur Respir J. 2016;10.1183/13993003.00893-2016. <http://erj.ersjournals.com/content/48/6/1682> [↑](#endnote-ref-11)
12. . Statistics Canada.  Table  36-10-0200-01   Historical: Personal expenditure on consumer goods and services at 1981 prices, 1968 System of National Accounts (SNA), annual, 1947 - 1986 (x 1,000,000) [↑](#endnote-ref-12)
13. . Statistics Canada.  Table  36-10-0225-01   Detailed household final consumption expenditure, provincial and territorial, annual (x 1,000,000) [↑](#endnote-ref-13)
14. . Statistics Canada.  Table  13-10-0660-01   Fruit and vegetable consumption, by age group and sex, household population aged 12 and over, Canada, provinces, territories and selected health regions (June 2005 boundaries) [↑](#endnote-ref-14)
15. . Bougie E, Kohen D. Smoking prevalence among Inuit in Canada. Health Reports. 2017 February; Statistics Canada Catalogue no. 82-003-X. <https://www150.statcan.gc.ca/n1/en/pub/82-003-x/2017002/article/14773-eng.pdf?st=DYMe5rG5> [↑](#endnote-ref-15)
16. . Statistics Canada.  Table  13-10-0451-01   Health indicators, annual estimates, 2003 - 2014 [↑](#endnote-ref-16)
17. . Ledrou I, Gervais J. Food Insecurity. Health Reports. 2005;16(3):47-51. Statistics Canada, Catalogue no. 82-003. <https://www150.statcan.gc.ca/n1/en/pub/82-003-x/2004003/article/7841-eng.pdf?st=vFM7I8SL> [↑](#endnote-ref-17)
18. . Egeland GM. IPY Inuit Health Survey speaks to need to address inadequate housing, food insecurity and nutrition transition. International Journal of Circumpolar Health. 2011;70(5):444-6. <https://www.tandfonline.com/doi/pdf/10.3402/ijch.v70i5.17854?needAccess=true> [↑](#endnote-ref-18)
19. . Arriagada P. Food insecurity among Inuit living in Inuit Nunangat**.** Insights on Canadian Society. 2017 February; Statistics Canada Catalogue no. 75-006-X. <https://www150.statcan.gc.ca/n1/en/pub/75-006-x/2017001/article/14774-eng.pdf?st=ls91FeEt> [↑](#endnote-ref-19)
20. . Tarasuk V, Mitchell A, Dachner N. Household Food Insecurity in Canada, 2014. Toronto. Research to identify policy options to reduce food insecurity (PROOF). 2016 [↑](#endnote-ref-20)
21. . Aboriginal Peoples in Canada in 2006: Inuit, Métis and First Nations, 2006 Census. Aboriginal Peoples, 2006 Census, Census year 2006. Statistics Canada Catalogue no. 97-558-XIE. <https://www12.statcan.gc.ca/census-recensement/2006/as-sa/97-558/pdf/97-558-XIE2006001.pdf> [↑](#endnote-ref-21)
22. . Census in Brief: The housing conditions of Aboriginal people in Canada. Census of Population, 2016. Statistics Canada Catalogue no. 98-200-X2016021. <https://www12.statcan.gc.ca/census-recensement/2016/as-sa/98-200-x/2016021/98-200-x2016021-eng.pdf> [↑](#endnote-ref-22)
23. . Statistics Canada. 2017. *Nunavut [Territory] and Canada [Country]* (table). *Census Profile*. 2016 Census. Statistics Canada Catalogue no. 98-316-X2016001. Ottawa. Released November 29, 2017.
    <https://www12.statcan.gc.ca/census-recensement/2016/dp-pd/prof/index.cfm?Lang=E> [↑](#endnote-ref-23)
24. . NUNAVUT DEPARTMENT OF HEALTH. Nunavut TB Manual 2017: NUNAVUT DEPARTMENT OF HEALTH; 2017. <https://www.gov.nu.ca/sites/default/files/nunavut_tb_manual_2017.pdf>. [↑](#endnote-ref-24)
25. . Centre for Communicable Diseases and Infection Control. Canadian Tuberculosis Standards. Public Health Agency, Canada. 2014;7th edition.[www.phac-aspc.gc.ca](file:///C:\Users\Olivia.Oxlade\Desktop\KS%20projects\Dynamic%20smoking%20model\BMC%20MEd%20Submission\www.phac-aspc.gc.ca) [↑](#endnote-ref-25)
26. . Pease C, Zwerling A, Mallick R, Patterson M, Demaio P, Finn S, Allen J, Van Dyk D, Alvarez GG. The latent tuberculosis infection cascade of care in Iqaluit, Nunavut, 2012–2016. BMC infectious diseases. 2019 Dec 1;19(1):890. <https://link.springer.com/content/pdf/10.1186/s12879-019-4557-3.pdf> [↑](#endnote-ref-26)
27. . Prochaska JJ, Delucchi K, Hall SM. A meta-analysis of smoking cessation interventions with individuals in substance abuse treatment or recovery. J Consult Clin Psychol. 2004;72(6):1144. <https://cloudfront.escholarship.org/dist/prd/content/qt0r8673wv/qt0r8673wv.pdf?t=lnrsdv> [↑](#endnote-ref-27)
28. . Stead LF, Perera R, Bullen C, Mant D, Hartmann-Boyce J, Cahill K, et al. Nicotine replacement therapy for smoking cessation. Cochrane Database Syst Rev. 2012;10.1002/14651858.CD000146.pub4(11). <http://dx.doi.org/10.1002/14651858.CD000146.pub4> [↑](#endnote-ref-28)
29. . Stead LF, Koilpillai P, Lancaster T. Additional behavioural support as an adjunct to pharmacotherapy for smoking cessation. Cochrane Database Syst Rev. 2015;10.1002/14651858.CD009670.pub3(10). <http://dx.doi.org/10.1002/14651858.CD009670.pub3> [↑](#endnote-ref-29)
30. . Marley JV, Atkinson D, Kitaura T, Nelson C, Gray D, Metcalf S, et al. The Be Our Ally Beat Smoking (BOABS) study, a randomised controlled trial of an intensive smoking cessation intervention in a remote aboriginal Australian health care setting. BMC public health. 2014;14(1):32 [↑](#endnote-ref-30)
31. . Levy DT, Chaloupka F, Gitchell J. The effects of tobacco control policies on smoking rates: a tobacco control scorecard. J Public Health Manag Pract. 2004;10(4):338-53. <http://ovidsp.tx.ovid.com/ovftpdfs/FPDDNCGCFCDFCG00/fs047/ovft/live/gv031/00124784/00124784-200407000-00011.pdf> [↑](#endnote-ref-31)
32. . Gallet CA, List JA. Cigarette demand: a meta-analysis of elasticities. Health Econ. 2002;12(10):821-35. <http://dx.doi.org/10.1002/hec.765> [↑](#endnote-ref-32)
33. .  Levy DT, Hyland A, Higbee C, Remer L, Compton C. The role of public policies in reducing smoking prevalence in California: results from the California tobacco policy simulation model. Health policy. 2007;82(2):167-85. <https://www.ncbi.nlm.nih.gov/pmc/articles/PMC2743269/pdf/nihms25327.pdf> [↑](#endnote-ref-33)
34. . Levy D, de Almeida LM, Szklo A. The Brazil SimSmoke policy simulation model: the effect of strong tobacco control policies on smoking prevalence and smoking-attributable deaths in a middle income nation. PLoS Med. 2012;9(11):e1001336. <https://www.ncbi.nlm.nih.gov/pmc/articles/PMC3491001/pdf/pmed.1001336.pdf> [↑](#endnote-ref-34)
35. . Indigenous Services Canada. ISC and Nunavut government partner to fund mobile addiction treatment program. Government of Canada. 2019 March. https://www.sac-isc.gc.ca/eng/1548425217756/1548425244542?wbdisable=true [↑](#endnote-ref-35)
36. . NVision Insight Group Inc. Summary Report: Addictions and Trauma Treatment in Nunavut. Government of Nunavut. 2018 August. <https://www.gov.nu.ca/sites/default/files/gn_att_executive_summary_summary_report_-_final_-_english.pdf> [↑](#endnote-ref-36)
37. . Kenny TA, Wesche SD, Fillion M, MacLean J, Chan HM. Supporting Inuit food security: A synthesis of initiatives in the Inuvialuit Settlement Region, Northwest Territories. Canadian Food Studies/La Revue canadienne des études sur l'alimentation. 2018 May 28;5(2):73-110. <https://canadianfoodstudies.uwaterloo.ca/index.php/cfs/article/view/213/281> [↑](#endnote-ref-37)
38. . Riva M, Fletcher C, Dufresne P, Perreault K, Muckle G, Potvin L, Bailie RS. Relocating to a new or pre-existing social housing unit: significant health improvements for Inuit adults in Nunavik and Nunavut. Canadian Journal of Public Health. 2020 Feb;111(1):21-30. <https://link.springer.com/content/pdf/10.17269/s41997-019-00249-6.pdf> [↑](#endnote-ref-38)
39. . Levy DT, Cummings KM, Hyland A. Increasing taxes as a strategy to reduce cigarette use and deaths: results of a simulation model. Prev Med. 2000;31(3):279-86 [↑](#endnote-ref-39)
40. . Wright S, Nebelkopf E, King J, Maas M, Patel C, Samuel S. Holistic System of Care: Evidence of Effectiveness. Substance Use & Misuse. 2011;46(11):1420-1430. [↑](#endnote-ref-40)
41. . Thompson S, Kamal AG, Alam MA, Wiebe J. Community Development to Feed the Family
    in Northern Manitoba Communities: Evaluating Food Activities Based on Their Food Sovereignty, Food Security, and Sustainable Livelihood Outcomes. Canadian Journal of Nonprofit and Social Economy Research. 2012;3(2):43-66. <https://anserj.ca/index.php/cjnser/article/view/121/67> [↑](#endnote-ref-41)
42. . Service des relations avec la clientèle. List of Medications. RAMQ, editor. Quebec: Bibliothèque et Archives nationales du Québec, 2017; 2017. 642 p<http://www.ramq.gouv.qc.ca/en/citizens/prescription-drug-insurance/Pages/prescription-drugs-covered.aspx> [↑](#endnote-ref-42)
43. . Pauktuutit Inuit Women of Canada. Facilitator’s Guide, Atii! Reduce Second-Hand Smoke. In: Pauktuutit Inuit Women of Canada, editor. Canada2012.<http://pauktuutit.ca/wp-content/blogs.dir/1/assets/Pauktuutit-ATII-FG-Phase2-English.pdf> [↑](#endnote-ref-43)
44. . Oxlade O, Sugarman J, Alvarez GG, Pai M, Schwartzman K. Xpert® MTB/RIF for the diagnosis of tuberculosis in a remote Arctic setting: Impact on cost and time to treatment initiation. PloS one. 2016;11(3):e0150119. <https://www.ncbi.nlm.nih.gov/pmc/articles/PMC4798714/pdf/pone.0150119.pdf> [↑](#endnote-ref-44)
45. . King BA, Pechacek TF, Mariolis P. Best practices for comprehensive tobacco control programs, 2014. 2014. <https://www.cdc.gov/tobacco/stateandcommunity/best_practices/pdfs/2014/comprehensive.pdf> [↑](#endnote-ref-45)
46. . Niqinik Nuatisivik Nunavut Food Bank. Inuit Tapiriit Kanatami: Nuluaq Mapping Project. 2018. <https://www.itk.ca/nuluaq-mapping-project/initiative/niqinik-nuatsivik-nunavut-food-bank/> [↑](#endnote-ref-46)
47. . The Canada Prenatal Nutrition Program: A Decade of Promoting the Health of Mothers, Babies and Communities. Public Health Agency of Canada, Division of Childhood and ADolescene, Centre for Health Promotion. 2007. <http://publications.gc.ca/collections/collection_2012/aspc-phac/HP10-11-2007-eng.pdf> [↑](#endnote-ref-47)
48. . Department of Economic Development and Transportation. 2015-16 Annual Report: Country Food Distribution Program. Government of Nunavut. 2017. <https://www.gov.nu.ca/sites/default/files/edt-2017-cfdp-ar_eng.pdf> [↑](#endnote-ref-48)
49. . Department of Economic Development and Transportation. 2018-2019 Programs and Funding. Government of Nunavut. 2019. <https://gov.nu.ca/sites/default/files/program_overview_pamphlet_eng_0.pdf> [↑](#endnote-ref-49)
50. . Inuit Tapiriit Kanatami. Inuit Nunangat Housing Strategy. Government of Canada. 2019 April; ISBN: 978-1-989179-25-3. <https://www.itk.ca/wp-content/uploads/2019/04/2019-Inuit-Nunangat-Housing-Strategy-English.pdf> [↑](#endnote-ref-50)
51. . Nunavut Housing Corporation. Nunavut Housing Corporation Annual Report 2016-17. Legislative Assembly of Nunavut. 2017;372-4(3). <https://assembly.nu.ca/sites/default/files/TD-372-4(3)-EN-NHC-2016-2017-Annual-Report_0.pdf> [↑](#endnote-ref-51)
52. . Tan M, Menzies D, Schwartzman K. Tuberculosis screening of travelers to higher-incidence countries: a cost-effectiveness analysis. BMC Public Health. 2008;8(1):201 [↑](#endnote-ref-52)
53. . Khan K, Meunnig P, Behta M, Zivin G. Global drug-resistance and the management of latent tuberculosis infection in immigrants to the United States. N Engl J Med. 2002 Dec;347(23):1850-9. <https://www.nejm.org/doi/full/10.1056/nejmsa021099> [↑](#endnote-ref-53)
54. . Ontario Ministry of Health and Long-Term Care. Schedule of Benefits, Physician Services Under the Health Insurance Act: Ministry of Health and Long-Term Care; 2015 May 1, 2015. <http://www.health.gov.on.ca/en/pro/programs/ohip/sob/physserv/sob_master11062015.pdf> [↑](#endnote-ref-54)
55. . Brown RE, Miller B, Taylor WR, Palmer C, Bosco L, Nicola RM, et al. Health-care expenditures for tuberculosis in the United States. Arch Intern Med. 1995 Aug;155(15):1595-600. [↑](#endnote-ref-55)
56. . Nunavut nurses: salary and bonuses. Iqaluit, NU, Canada: Government of Nunavut. 2008. <http://www.nunavutnurses.ca/english/jobs/salary_bonuses.shtml>. [↑](#endnote-ref-56)
57. . Sugarman J, Alvarez G, Schwartzman K, Oxlade O. Sputum induction for tuberculosis diagnosis in an Arctic setting: a cost comparison. Int J Tuberc Lung Dis. 2014;18(10):1223-30. [↑](#endnote-ref-57)
58. . Smith BM, Schwartzman K, Bartlett G, Menzies D. Adverse events associated with treatment of latent tuberculosis in the general population. CMAJ. 2011 Feb;183(3):E173-9. doi: <https://dx.doi.org/10.1503%2Fcmaj.091824> [↑](#endnote-ref-58)
59. . Brassard P, Anderson KK, Schwartzman K, Macdonald ME, Menzies D. Challenges to tuberculin screening and follow-up in an urban Aboriginal sample in Montreal, Canada. J Health Care Poor Underserved. 2008 May;19(2):369-79. doi: <https://doi.org/10.1353/hpu.0.0029> [↑](#endnote-ref-59)
60. . Comstock GW, Edwards LB, Livesay VT. Tuberculosis Morbidity in the US Navy: Its Distribution and Decline 1, 2. Am Rev Respir Dis. 1974;110(5):572-80 [↑](#endnote-ref-60)
61. . Nolan CM, Elarth AM. Tuberculosis in a cohort of Southeast Asian refugees. Am Rev Respir Dis. 1988;137:805-9 [↑](#endnote-ref-61)
62. . Behr MA, Edelstein PH, Ramakrishnan L. Revisiting the timetable of tuberculosis. BMJ. 2018;362:k2738.<https://www.bmj.com/content/bmj/362/bmj.k2738.full.pdf> [↑](#endnote-ref-62)
63. . Ditkowsky JB, Schwartzman K. Potential cost-effectiveness of a new infant tuberculosis vaccine in South Africa--implications for clinical trials: a decision analysis. PLoS One. 2014 Jan;9(1):e83526. doi: <https://doi.org/10.1371/journal.pone.0083526> [↑](#endnote-ref-63)
64. . Statistics Canada. 2012. Nunavut (Code 62) and Canada (Code 01) (table). Census Profile. 2011 Census. Statistics Canada Catalogue no. 98-316-XWE. Ottawa. Released October 24, 2012.
    <http://www12.statcan.gc.ca/census-recensement/2011/dp-pd/prof/index.cfm?Lang=E> [↑](#endnote-ref-64)
65. . Lin H-H, Ezzati M, Murray M. Tobacco smoke, indoor air pollution and tuberculosis: a systematic review and meta-analysis. PLoS Med. 2007;4(1):e20. <https://www.ncbi.nlm.nih.gov/pmc/articles/PMC1769410/pdf/pmed.0040020.pdf> [↑](#endnote-ref-65)
66. . Lin H-H, Murray M, Cohen T, Colijn C, Ezzati M. Effects of smoking and solid-fuel use on COPD, lung cancer, and tuberculosis in China: a time-based, multiple risk factor, modelling study. The Lancet. 2008;372(9648):1473-83 [↑](#endnote-ref-66)
67. . Borgdorff MW, Veen J, Kalisvaart NA, Nagelkerke N. Mortality among tuberculosis patients in The Netherlands in the period 1993-1995. Eur Respir J. 1998 Apr;11(4):816-20. <https://erj.ersjournals.com/content/erj/11/4/816.full.pdf> [↑](#endnote-ref-67)
68. . Fox GJ, Lee RS, Lucas M, Khan FA, Proulx JF, Hornby K, et al. Inadequate Diet Is Associated with Acquiring Mycobacterium tuberculosis Infection in an Inuit Community: A Case-Control Study. Ann Am Thorac Soc. 2015 Aug;12(8):1153-62. <https://www.atsjournals.org/doi/full/10.1513/AnnalsATS.201503-156OC?url_ver=Z39.88-2003&rfr_id=ori%3Arid%3Acrossref.org&rfr_dat=cr_pub%3Dpubmed#readcube-epdf> [↑](#endnote-ref-68)
69. . Berhe G, Enquselassie F, Aseffa A. Assessment of risk factors for development of active pulmonary tuberculosis in northern part of ethiopia: a matched case control study. Ethiop Med J. 2013 Oct;51(4):227-37. [↑](#endnote-ref-69)
70. . Kilabuk E, Momoli F, Mallick R, Van Dyk D, Pease C, Zwerling A, et al. Social determinants of health among residential areas with a high tuberculosis incidence in a remote Inuit community. J Epidemiol Community Health. 2019 May;73(5):401-406. <https://jech.bmj.com/content/jech/73/5/401.full.pdf> [↑](#endnote-ref-70)
71. . Khan FA, Fox GJ, Lee RS, Riva M, Benedetti A, Proulx JF, et al. Housing and tuberculosis in an Inuit village in northern Quebec: a case–control study. CMAJ Open. 2016;4(3):E496–E506. <https://www.ncbi.nlm.nih.gov/pmc/articles/PMC5047805/pdf/cmajo.20160049.pdf> [↑](#endnote-ref-71)
72. . Bauer M, Ahmed S, Benedetti A, Greenaway C, Lalli M, Leavens A, et al. Health-related quality of life and tuberculosis: a longitudinal cohort study. Health Qual Life Outcomes. 2015;13(1):65 [↑](#endnote-ref-72)
73. . Guo N, Marra CA, Marra F, Moadebi S, Elwood RK, FitzGerald JM. Health state utilities in latent and active tuberculosis. Value in health. 2008;11(7):1154-61 [↑](#endnote-ref-73)
